# A stochastic model of acute-care decisions based on patient and provider heterogeneity

**DOI:** 10.1007/s10729-015-9347-x

**Published:** 2015-10-21

**Authors:** Muge Capan, Julie S. Ivy, James R. Wilson, Jeanne M. Huddleston

**Affiliations:** 1 Value Institute, Christiana Care Health System, John H. Ammon Medical Education Center, 4755 Ogletown-Stanton Road, Newark, DE 19718 USA; 2 Edward P. Fitts Department of Industrial and Systems Engineering, North Carolina State University, Campus Box 7906, Raleigh, NC 27695-7906 USA; 3 Robert D. and Patricia E. Kern Center for the Science of Health Care Delivery, Mayo Clinic, 200 First Street SW, Rochester, MN 55905 USA

**Keywords:** Acute physiological deterioration, Early warning scores, Semi-Markov decision process model, Cluster analysis, 60J28 (Applications of continuous-time Markov processes on discrete state spaces)

## Abstract

The primary cause of preventable death in many hospitals is the failure to recognize and/or rescue patients from acute physiologic deterioration (APD). APD affects all hospitalized patients, potentially causing cardiac arrest and death. Identifying APD is difficult, and response timing is critical - delays in response represent a significant and modifiable patient safety issue. Hospitals have instituted rapid response systems or teams (RRT) to provide timely critical care for APD, with thresholds that trigger the involvement of critical care expertise. The National Early Warning Score (NEWS) was developed to define these thresholds. However, current triggers are inconsistent and ignore patient-specific factors. Further, acute care is delivered by providers with different clinical experience, resulting in quality-of-care variation. This article documents a semi-Markov decision process model of APD that incorporates patient and provider heterogeneity. The model allows for stochastically changing health states, while determining patient subpopulation-specific RRT-activation thresholds. The objective function minimizes the total time associated with patient deterioration and stabilization; and the relative values of nursing and RRT times can be modified. A case study from January 2011 to December 2012 identified six subpopulations. RRT activation was optimal for patients in “slightly concerning” health states (NEWS > 0) for all subpopulations, except surgical patients with low risk of deterioration for whom RRT was activated in “concerning” states (NEWS > 4). Clustering methods identified provider clusters considering RRT-activation preferences and estimation of stabilization-related resource needs. Providers with conservative resource estimates preferred waiting over activating RRT. This study provides simple practical rules for personalized acute care delivery.

## Introduction

Patient physiology can change unpredictably and dynamically over the course of a hospitalization. Every patient who is admitted to the hospital is at risk of experiencing *acute physiological deterioration* (APD), defined as acute and persistent abnormality in one or multiple physiological measures, potentially resulting in cardiac arrest, unscheduled intensive care unit (ICU) admission, or death. Major challenges are that APD is difficult to identify, response timing is critical, current measures for evaluating patient condition are not consistent, and rules for involvement of critical care expertise are ignoring patient characteristics.

Patient rescue is a complex problem. The acute care of patients experiencing APD relies on early recognition and rapid response to stabilize the patient’s condition. Medical emergency/rapid response systems are composed of periodic monitoring of physiologic status with critical thresholds that trigger the involvement of early critical care expertise during APD with the goal of preserving health and preventing undesired health outcomes. Early Warning Systems (EWSs) are quantitative scoring systems that assign values to selected physiological measures to detect abnormalities and inform clinical decision making in cases of APD [1, 2]. However, there is little standardization with respect to the best use of these scores and current critical thresholds are based primarily on subjective clinical judgement.

Physiological deterioration is characterized by notable changes in vital signs. Routinely collected vital signs can inform recognition of these changes. Increasing complexity of care delivery processes and the fragmented nature of health care delivery are some of the main challenges that lead to lack of or delayed recognition and response to APD. Failure to (or delay in) recognize and respond to APD remains a challenge for health care systems. The National Patient Safety Agency reports that 11 % of serious hospital incidents arise through failure to act on deterioration, with failure to recognize the importance of physiological deterioration as one of the primary reasons [3].

The uncertainty in physiological deterioration and recovery processes during hospitalization can be modeled as a sequential decision problem under uncertainty. EWS-based dynamic decision models can inform acute-care practice at the point of care delivered by medical emergency teams with critical care expertise, such as a *Rapid Response Team* (*RRT*) [4, 5]. Clinical guidelines classify physiological condition using EWSs and suggest RRT activation for scores above critical thresholds [1]. Predictive performance of EWSs for undesired incidents during hospitalization is well studied; however, several gaps remain in the literature [6–11]. The individualized use of EWSs to identify personalized resuscitation interventions, such as RRT activation, is a natural extension of this literature with a focus on the impact of patient-specific risk.

While RRTs have been widely implemented in health care systems, evidence to support their effectiveness is insufficient [12]. Several studies focused primarily on patient outcomes to assess the RRT effectiveness; however, the findings are mixed [13–15]. A major multi-center controlled trial (the Medical Early Response Intervention and Therapy (MERIT)) study investigated whether RRT implementation reduces the incidence of cardiac arrests, unplanned admissions to intensive ca re units (ICU), and deaths [13]. The study was unable to provide sufficient evidence to demonstrate effectiveness of RRTs. The results of the study showed that RRT implementation was not associated with a decrease in cardiac arrests, ICU admissions, or deaths. Chan et al. (2010) conducted a meta-analysis of 18 studies (1950 through 2008) to assess the effect of RRTs on reducing cardiopulmonary arrest and hospital mortality rates, and reported mixed results [14]. While some studies showed a reduction in cardiopulmonary arrest rates in adults outside the ICU, the reductions were not associated with lower hospital mortality rates. Jones et al. (2009) reviewed several studies to assess whether RRT dose (i.e., RRT calls per 1,000 patient admissions or discharges) impacts patient outcomes [15]. The findings suggested a greater effect in patient outcomes (e.g., reduction in the rate of cardiac arrests) with a greater dose of care from RRT. Leach and Mayo (2013) conducted a qualitative analysis of RRT effectiveness and identified RRT management challenges including inconsistency of team members from day to day, limited opportunity for RRT members to develop team skills, and greater need for team training compared to clinical teams that work together regularly under less time pressure to perform [12]. In conclusion, measurement of RRT effectiveness and management remains a challenge, and quantitative metrics focusing on earlier recognition of physiological decline and better utilization of limited RRT resources may support capturing the impact of RRT implementation in clinical practice.

An effective EWS-based decision model needs to address the impact of *both patient and provider heterogeneity* to accurately capture the dynamics of APD and identify optimal RRT-activation policies. APD impacts the *heterogeneous patient population* with different reasons for admission and different clinical trajectories. Existing EWSs ignore this heterogeneity. For example, existing RRT activation criteria are the same for all patients and do not differentiate the intervention based on patient characteristics. Further, neglecting patient heterogeneity may translate to unnecessary activation of RRT, resulting in suboptimal use of limited personnel resources, and inaccurate estimation of resource needs.

In addition to patient heterogeneity, acute care is delivered by diverse care provider teams including physicians, critical care nurses, and respiratory therapists. The team composition may differ by facility and may be subject to changes over time within the same facility. *Heterogeneity in provider teams* may result in clinical practice variation, impacting the evaluation of patient resource needs. This variation may impact RRT activation decisions. Understanding the dynamics of APD associated with heterogeneity at the patient and provider levels and the stochastic nature of the health system is necessary to foster personalized medical decision making.

In this article, we develop a semi-Markov decision process (SMDP) model for the management of a patient’s physiological condition. The model allows for stochastically changing health states (the main source of uncertainty) while determining patient subpopulation-specific National Early Warning Score (NEWS)-based RRT activation thresholds. The objective is to minimize the total time associated with patient deterioration and stabilization, including the times associated with clinical distress from the providers’ (decision maker’s) perspective, nursing activities, RRT activities, and stabilization. Further, to incorporate the impact of provider heterogeneity we develop a modified SMDP model that examines the worst-case scenarios in terms of nursing and RRT time (by weighting these times) and using the maximum time (instead of actual times) of nurse and RRT resources to associate a relative value between the two types of resources. The goal of modifying the nursing and RRT times and the relative values between nursing and RRT effort is to represent provider heterogeneity in the response to APD. Using clustering methods, we identify categories of providers with distinct RRT-activation preferences and distinct valuation of resource needs of patients.

### Main contributions of this article

The main contributions of this article are the following: (i) formulation and implementation of a stochastic decision model that explicitly takes into account both patient and provider heterogenity; and (ii) formulation and evaluation of optimal personalized RRT-activation policies and total expected stabilization-related time. We have analyzed large datasets containing over 50,000 patient records to identify clinically relevant patient subpopulations and populate the SMDP model. Considering the heterogeneity and uncertainty in acute-care systems, including patients and providers, we hypothesize that the differences in provider valuation of RRT resource requirements and patient nursing needs influence the RRT activation decision making. However, we hypothesize that clusters of providers may be identified which have common RRT strategies.

An outline of the remainder paper is as follows. First, an overview of the relevant literature is presented. Using findings from our previous work, we present the baseline SMDP model illustrated on a retrospective case study including 55,385 patients hospitalized at a single facility (self-identifying name of the facility omitted) from January 2011 to December 2012. Next, provider heterogeneity is modeled in the modified SMDP model. We discuss the findings and their implications.

### Previous literature on SMDP models in health care

A Markov Decision Process (MDP) model provides a framework for representing multi-stage decision problems in the presence of uncertainty. Markovian models are commonly used in health care to support screening, diagnosis, and treatment decisions for chronic conditions, patient flow, and hospital operations optimization [16–19]. For continuous-time MDPs, the Markov property ensures that the time spent in a state before a transition occurs, i.e., the *sojourn time*, follows an exponential distribution. However, in cases of physiological deterioration and recovery, the sojourn time may not be exponentially distributed. A semi-Markov decision process (SMDP) model is a generalization of an MDP in which the state transitions follow a Markov chain; however, the sojourn time is a random variable following an arbitrary distribution [20–22]. SMDP models have been applied in health care since the early 1970s to model the following: (i) resource planning and personnel scheduling within hospitals [23, 24]; (ii) modeling patient condition during hospitalization such as recovery processes of acute leukemia patients [25], coronary patients [26], and end-stage renal disease patients [27]; and (iii) patient flow in a maternity service unit [28]. Unfortunately, this research ignores patient and provider heterogeneity as well as the impact of the sojourn time and its role in clinical decision making. Further, (self-identifying reference omitted) used a National Early Warning Score (NEWS)-based continuous-time SMDP model to develop subpopulation-specific resuscitation thresholds [29]. However, their study did not consider provider heterogeneity, did not assign weights to time-based metrics and their relative values, did not differentiate between different types of stabilization, and did not use clustering methods. Our study bridges this gap in the literature to capture patient heterogeneity by identifying subpopulations based on patient characteristics, and using optimization and clustering methods to incorporate provider heterogeneity.

## Methods

### Identifying patient subpopulations

Subpopulations within a heterogeneous patient population are identified to enable patient-centered decision making. Our methodology for selecting patient characteristics to identify statistically significantly different subpopulations is described in (self-identifying reference omitted), who identified two patient characteristics for classifying subpopulations: risk of deterioration (ROD) during hospitalization (i.e., low, moderate or high ROD based on the Braden skin score, which is a risk assessment tool commonly used at admission); and admission type (i.e., medical or surgical) [29]. Medical admission refers to the patients admitted for symptoms of discomfort or illness and admitted for a reason other than surgery. Surgical patients are admitted for a surgical procedure. In this article, we use six subpopulations defined by ROD and admission type:

- subpopulation (A) – medical patients with low ROD;
- subpopulation (B) – surgical patients with low ROD;
- subpopulation (C) – medical patients with moderate ROD;
- subpopulation (D) – surgical patients with moderate ROD;
- subpopulation (E) – medical patients with high ROD; and
- subpopulation (F) – surgical patients with high ROD.

**Fig. 1:**
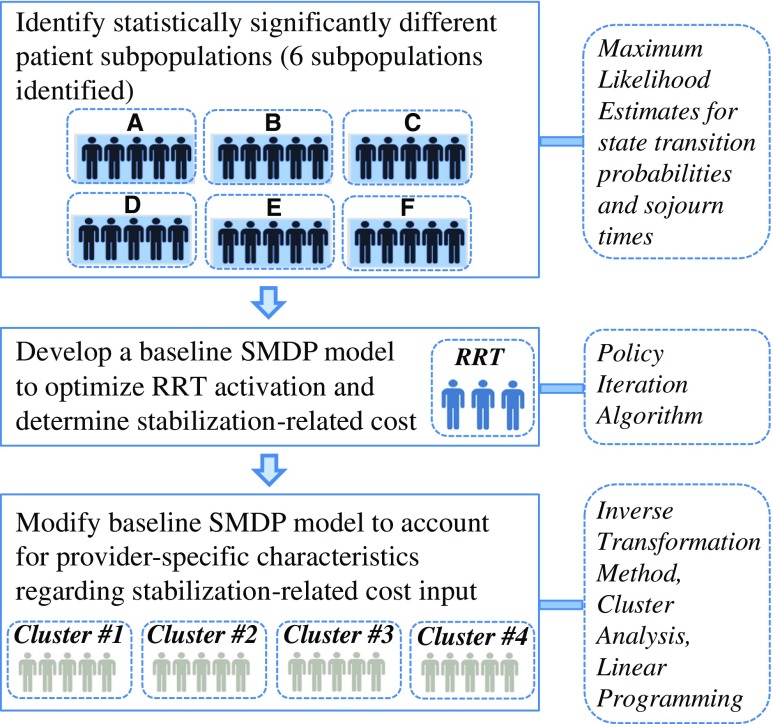
Methodological approach for stochastic acute-care decision optimization considering patient and provider heterogeneity. The *squares* on the left represent each modeling step, and *boxes* with dashed borders on the right represent corresponding methods

Figure 1 summarizes our methodological approach.

### Baseline SMDP model

The baseline SMDP model, defined by the set of elements (*S*, *T*, *A*, *P*, *r*, *F*), represents the progress of a patient’s physiological condition as a continuous-time stochastic process: a finite set of health states *S*, an infinite time horizon *T*, a finite set of actions *A*, state transition probability *P* (defining probablistic movement between states conditional on the current state and action), stabilization and resource time *r*(*s*, *d*(*s*)) associated with performing the action *d*(*s*) in state *s*, and the sojourn time cumulative distribution function, *F*(*t*|*s*, *d*(*s*)) for a patient in state *s* and when action *d*(*s*) is taken. For a given stationary policy *π* in the set of *Π* of feasible policies, the minimum total expected discounted stabilization and resource time for health state *s* is given by the value function

$$ {v}_{\alpha, {\pi}^{*}}(s)\equiv { \min}_{\pi \in \varPi \kern0.5em }\left\{{v}_{\alpha, \pi }(s)\right\}, $$

where

$$ {v}_{\alpha, \pi }(s)=r\left(s,d(s)\right)+{\displaystyle {\sum}_{j\in S}P\left(j|s,d(s)\right){\displaystyle {\int}_0^{\infty }{e}^{-\alpha t}F\left(dt|s,\ d(s)\right)\ {v}_{\alpha, \pi }(j)}}, $$

And π * denotes the optimal stationary policy. We use continuous discounting denoted by *e* ^− *at*^ with *α* = 0.03 corresponding to a commonly used discrete discounting factor [30].

#### Decision epochs

The stochastic process starts at the beginning of a *hospitalization episode*. This can be a general ward admission, or a return to the general ward from a higher-level care unit. A *decision epoch* is defined as a point in time corresponding to a provider team assessment during regular hospital rounding that identifies a change in the patient’s health condition. Thus, a new decision epoch in the continuous-time stochastic model occurs only when the patient’s health condition differs from the immediately preceding evaluation.

#### Health states

Health states focus on features of the patient’s condition. The set of states *S* is the same for all six subpopulations. The core continuous-time stochastic process, {*X*_*t*_, *t* ≥ 0} represents the patient’s current health state measured by the NEWS [1]. Model states *X*_*t*_ = *s* ∈ *S* = {1, 2, 3, 4*f*, 4*s*, 5, 6} are ordered such that a lower value of NEWS is associated with better health (Table 1). The classification of NEWS values and their clinical interpretation in Table 1 are derived from clinical guidelines [1].

**Table 1:** Health states in the baseline SMDP and the corresponding clinical interpretation [1]

NEWS	Health state	Clinical interpretation
–	6	Discharged from the general ward
0	5	Stable (NEWS has not exceeded 0 since start of hospitalization episode)
0	4s	Stabilized after deterioration, i.e., maintained NEWS=0 for ≥ 1 h
0	4f	Returned to NEWS=0 after deterioration
1−4	3	Slightly concerning
5−6 or individual physiological parameter with significant abnormality	2	Concerning
≥7	1	Distress

The states {4*f*,4s,6} are model extensions of clinical guidelines to capture stabilization and discharge conditions. State 6 includes all outcomes that result in the patient leaving the general ward, including transfer to a higher-level care, discharge alive, admission to hospice, and death. State 5 represents a patient who has not been observed with NEWS >0 since the start of the current hospitalization episode. State 4*f* represents returning to NEWS =0 following APD. Based on expert medical opinion, we assume that maintaining NEWS =0 for at least 1 h following APD represents stabilization, i.e., remaining in state 4*f* for at least 1 h results in a movement from 4*f* to the stabilized state 4*s*.

#### Actions

Care provider actions are allowed only at decision epochs (i.e., are prompted by changes in patient condition) and include *waiting*, i.e., *d*(*s*) = 1, postponing the RRT activation until the next clinical assessment, or *activating the RRT* immediately, i.e., *d*(*s*) = 2. Waiting refers to the case when the bedside provider decides to provide necessary acute care for the patient without using critical care (RRT) resources for guidance. Activating RRT refers to the case when the bedside provider decides to obtain critical care guidance by calling the RRT immediately. Waiting is allowed in all states, whereas activating the RRT is allowed only in states {1,2,3} , which are associated with NEWS > 0. The state-dependent action space is chosen for the following reasons: (i) the historical data did not show any RRT activation in states {4*f*,4*s*,5}; (ii) the stabilized condition of patients with NEWS =0 typically does not need bedside critical care evaluation; and (iii) activating the RRT in discharged state 6 is not possible because RRT provides critical care only for the patients in the general ward.

#### Transition probabilities

**Fig. 2:**
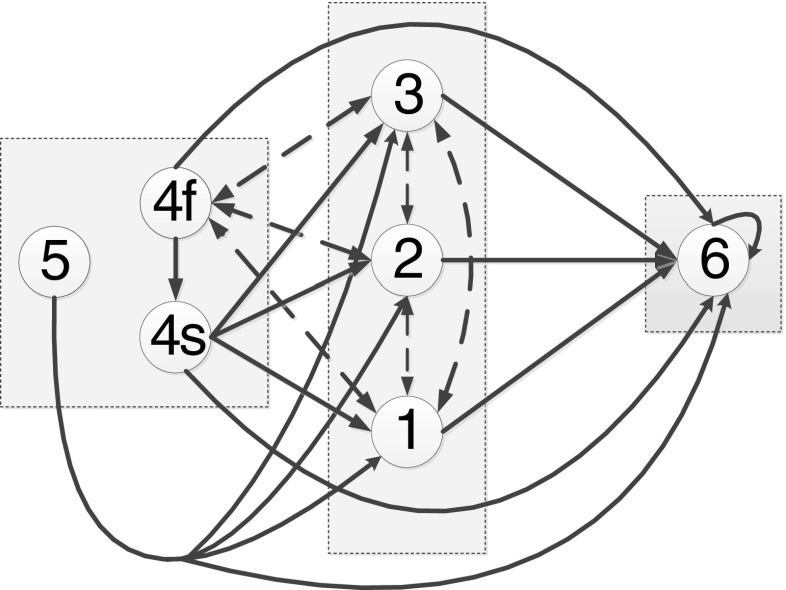
State transition diagram showing possible movements between model states

The state transition probabilities govern changes in health state as a function of the subpopulation. Figure 2 illustrates the possible state transitions.

In Fig. 2, the patient’s health states are classified into three groups: states {4*f*,4*s*,5} corresponding to NEWS =0, states {1,2,3} with NEWS >0 and an absorbing state 6. Dashed arrows are bidirectional state movements, and solid arrows represent unidirectional state movements. For example, given the current state is 1, the next state is in the set {2,3,4*f*,6}. Because 6 is an absorbing state, it is not possible to leave state 6, so the arrow 1 → 6 is solid, whereas the arrow from state 1 to a state in {2,3,4*f*} is dashed, i.e., state movements 1 → {2,3,4*f*} → 1 are allowed.

#### Sojourn times

We assume that the patient’s physiologic condition is observed retrospectively at decision epochs. Owing to changes in the patient’s physiological condition that are observed via irregular monitoring of patients in the general ward, the time between decision epochs is a random variable. Sojourn times in the SMDP model are estimated from empirical distributions conditional on subpopulation, current state, action, and next state, as derived from electronic medical records (EMRs) (Appendix 1, Tables 6, 7, 8, 9, 10, 11, 12, 13, 14, 15, 16 and 17).

#### Stabilization and resource time parameters

The stabilization and resource time accumulated between two decision epochs, *r*(*s*,*d*(*s*)), includes the following components: *γ*_nurse_(*s*); *γ*_RRT_(*s*), *Time to Stabilization* (*TTS*) and *Failure to Rescue* (*FTR*). The nurse time, *γ*_nurse_(*s*), refers to the average additional time in hours to provide care for a patient in health states as derived from the nursing records provided by (self-identifying name of the facility omitted) compared with average nursing needs in state 5. The term *γ*_RRT_(*s*) is the average RRT resource time in hours from activation until departure from the patient’s bedside, given the patient was in state *s* at the time of RRT activation. We assume that no RRT and nurse times are accumulated after discharge for the purposes of modeling APD in the general ward.

TTS refers to time from the start of APD until the patient is stabilized. Start of APD is defined by expert medical opinion as the point in time when the deviation from the normal range for any physiological measure included in NEWS exceeds 30 min. Therefore, TTS includes the following:

- recovery (i.e., the current state is *s* ∈ {1, 2, 3}, and next state is *j* = {4*f*});
- successful stabilization (i.e., movement out of the current state *s* = {4*f*} occurs after 1 h so that next state is *j* = {4*s*}); and
- unsuccessful stabilization (i.e., movement out of the current state *s* = {4*f*} occurs in less than 1 h, and next state is *j* ∈ {1, 2, 3, 6}).

FTR refers to the case when NEWS remains ≥ 7 (i.e., the patient is in distress or model state 1) for at least 1 h without RRT activation. Current NEWS guidelines recommend that: (i) a NEWS value of 5–6 should trigger a medium-level clinical alert, i.e., an urgent clinical review; (ii) a NEWS value of 7 or more should trigger a high-level clinical alert, e.g., an RRT activation, and (iii) an extreme weight in any one physiological parameter should trigger a medium-level alert [1].

The objective of the baseline SMDP model is to minimize the sum of the stabilization time (TTS), time in a critical clinical condition (FTR), and resource use (including nurse and RRT time). Final expressions of the stabilization and resource time functions are presented below. The function, *r*(*s*,*d*(*s*)), is given by:

1a $$ r\left(s,d(s)\right)=0\kern1.25em \mathrm{f}\mathrm{o}\mathrm{r}\ \mathrm{s}\in \left\{5,\ 4\mathrm{s}\right\},\mathrm{d}\left(\mathrm{s}\right)=\left\{1\right\}, $$

1b $$ \begin{array}{l}r\left(s,d(s)\right) = {\gamma}_{\mathrm{nurse}}(s)+I\left(d(s)=2\right)\ {\gamma}_{\mathrm{RRT}}(s)\hfill \\ {}+{\displaystyle {\int}_0^{\infty}\left[{\displaystyle {\int}_0^u{e}^{-\alpha t}\cdot 1 \cdot P\left(4f|s,d(s)\right)dt}\right]}\ {\lambda}_{s,d(s)}{e}^{-{\lambda}_{s,d(s)}u}du\hfill \\ {}\mathrm{f}\mathrm{o}\mathrm{r}\ \mathrm{s}\in \left\{2,\ 3\right\},\ \mathrm{j}=\left\{4\mathrm{f}\right\},\kern0.5em \mathrm{d}\left(\mathrm{s}\right)\in \left\{1,\ 2\right\},\hfill \end{array} $$

1c $$ \begin{array}{l}r\left(s,d(s)\right) = {\gamma}_{\mathrm{nurse}}(s)\hfill \\ {}+{\displaystyle {\int}_0^{\infty}\left[{\displaystyle {\int}_0^u{e}^{-\alpha t}\cdot 1 \cdot P\left(4f|s,d(s)\right)dt}\right]}{\lambda}_{s,d(s)}{e}^{-{\lambda}_{s,d(s)}u}du\hfill \\ {}+{\displaystyle {\int}_1^{\infty }{\displaystyle {\sum}_{j\in S}}\left[{\displaystyle {\int}_0^u{e}^{-\alpha t}\cdot 1 \cdot P\left(j|s,d(s)\right)dt}\right]{\lambda}_{s,d(s)}{e}^{-{\lambda}_{s,d(s)}u}du}\hfill \\ {}\mathrm{f}\mathrm{o}\mathrm{r}\ \mathrm{s}\in \left\{1\right\},\ \mathrm{j}\in \mathrm{S},\ \mathrm{d}\left(\mathrm{s}\right)=\left\{1\right\},\hfill \end{array} $$

1d $$ \begin{array}{l}r\left(s,d(s)\right) = {\gamma}_{\mathrm{nurse}}(s) + {\gamma}_{\mathrm{RRT}}(s)\hfill \\ {}+{\displaystyle {\int}_0^{\infty}\left[{\displaystyle {\int}_0^U{e}^{-\alpha t}\cdot 1\cdot P\left(4f|s,d(s)\right)dt}\right]{\lambda}_{s,d(s)}{e}^{-{\lambda}_{s,d(s)}u}du}\hfill \\ {}\mathrm{f}\mathrm{o}\mathrm{r}\ \mathrm{s}\in \left\{1\right\},\ \mathrm{j}=\left\{4\mathrm{f}\right\},\kern0.5em \mathrm{d}\left(\mathrm{s}\right)=\left\{2\right\},\hfill \end{array} $$

1e $$ \begin{array}{l}r\left(s,d(s)\right) = {\gamma}_{\mathrm{nurse}}(s)\hfill \\ {}+{\displaystyle {\int}_0^1{\displaystyle {\sum}_{j\in \left\{1,2,3,6\right\}}P\left(j|4f,\ d(4f),Z<1\right)}}\left[{\displaystyle {\int}_0^u{e}^{-\alpha t}\cdot 1\cdot dt}\right]{\lambda}_Z{e}^{-{\lambda}_Zu}du\hfill \\ {} + {e}^{-{\lambda}_Z}{\displaystyle {\int}_0^1{e}^{-\alpha t}\cdot 1\cdot dt}\hfill \\ {}\mathrm{f}\mathrm{o}\mathrm{r}\ \mathrm{s}\in \left\{4\mathrm{f}\right\},\ \mathrm{d}\left(\mathrm{s}\right)\in \left\{1\right\}\hfill \end{array} $$

where: (i) *I*(*d*(*s*) = 2) is the indicator function for the condition in the outermost parentheses so that *I*(*d*(*s*) = 2) = 1 if the condition *d* (*s*) = *2* is true, and *I*(*d*(*s*) = 2)=0 if *d*(*s*) ≠ 2; (ii) the term *λ*_*s*,*d*(*s*)_ is the state- and action-dependent rate parameter for the sojourn time distribution in state *s* until the next decision epoch; (iii) *λ*_*s*,*d*(*s*)_ = ∑_*j* ∈ *S*,*j* ≠ *s*_ *λ*_*s*,*d*(*s*),*j*_ where *λ*_*s*,*d*(*s*),*j*_ is the rate corresponding to the exponential time spent in *s* before a transition to *j* would occur, given action *d*(*s*); and (iv) *Z* = min {*H*_4*f*,*d*(4*f*),*j*_ : *j* = {1, 2, 3, 6}} is exponential with rate parameter *λ*_*Z*_ = ∑_*j* ∈ (1,2,3,6)_ *λ*_4*f*,*d*(4*f*),*j*_ where *H*_4*f*,*d*(4*f*),*j*_ represents the potential time to make the transition 4*f* → *j*. Derivation of the expressions of the stabilization and resource time are discussed in detail in Appendices 2, 3 and 4.

### Modified SMDP model

Most of the literature on MDPs assumes that the model parameters such as state transition probabilities and costs are known to the decision maker [31]. However, in practice, the model parameters must be estimated from data. For Markovian models, deriving model inputs from data may cause errors in estimation of model parameters [32]. Several studies address uncertainty in state transition probabilities [31, 33, 34]. Fewer studies focus on uncertainty in cost [32, 35]. In our study, because time is critical in the response to APD, time is considered as the “cost”. The acute-care provider team may consist of different providers and the team composition dynamically changes over time. In addition, providers may have different degrees of clinical experience and there may be variations in response to APD from one team to the next. It is particularly important to account for variation in the providers’ valuation of stabilization and resource time associated with health states and actions. The modified SMDP model incorporates the following: (i) the classification of care providers into *clusters* having similar characteristics; and (ii) the determination of the impact of provider heterogeneity on optimal RRT-activation policy as well as stabilization and resource time.

#### Provider heterogeneity parameters

Provider heterogeniety is captured by identifying groups of providers with similar time perception characteristics, defined as *clusters*. We introduce two state-dependent model parameters: *ρ*(*s*) and *ξ*(*s*). In the modified SMDP model, providers assign weights to average and maximum nurse and RRT times, with the maximum nurse and RRT times representing the worst-case scenario. The parameter *ρ*(*s*) takes values in [0, 1] and the stabilization and resource time function *r*′(*s*, *d*(*s*)), given state *s* and action *d*(*s*), is:

$$ {r}^{\prime}\left(s,d(s)\right)=\rho (s)r\left(s,d(s)\right) + \left(1-\rho (s)\right)\overline{r}\left(s,d(s)\right). $$

The term $\overline{r}\left(s,d(s)\right)$ is the “worst-case” stabilization and resource-time function in which the average additional nurse time and average RRT resource time are replaced with the corresponding maximum values for each model state as derived from EMRs. The term *r*(*s*, *d*(*s*)) is as defined in Eq. (1a–e). The final expressions for $\overline{r}\left(s,d(s)\right)$ are presented below . The function $\overline{r}\left(s,d(s)\right)$ is given by:

$$ \overline{r}\left(s,d(s)\right)={\overline{\gamma}}_{\mathrm{nurse}}(s)\kern1.5em \mathrm{f}\mathrm{o}\mathrm{r}\ \mathrm{s}\in \left\{5,\ 4\mathrm{s}\right\},\mathrm{d}\left(\mathrm{s}\right)=\left\{1\right\}, $$

$$ \begin{array}{l}\overline{r}\left(s,d(s)\right)={\overline{\gamma}}_{\mathrm{nurse}}(s)+I\left(d(s)=2\right){\overline{\gamma}}_{\mathrm{RRT}}(s)\hfill \\ {}+{\displaystyle {\int}_0^{\infty}\left[{\displaystyle {\int}_0^u{e}^{-\alpha t}\cdot 1\cdot P\left(4f|s,d(s)\right)dt}\right]}{\lambda}_{s,d(s)}{e}^{-{\lambda}_{s,d(s)}u}du\hfill \\ {}\mathrm{f}\mathrm{o}\mathrm{r}\ \mathrm{s}\in \left\{2,\ 3\right\},\ \mathrm{j}=\left\{4\mathrm{f}\right\},\ \mathrm{d}\left(\mathrm{s}\right)\in \left\{1,\ 2\right\},\hfill \end{array} $$

$$ \begin{array}{l}\overline{r}\left(s,d(s)\right)={\overline{\gamma}}_{\mathrm{nurse}}(s)\hfill \\ {}+{\displaystyle {\int}_0^{\infty}\left[{\displaystyle {\int}_0^u{e}^{-\alpha t}\cdot 1\cdot P\left(4f|s,d(s)\right)dt}\right]}{\lambda}_{s,d(s)}{e}^{-{\lambda}_{s,d(s)}u}du\hfill \\ {}+{\displaystyle {\int}_1^{\infty }{\displaystyle {\sum}_{j\in S}\left[{\displaystyle {\int}_0^u{e}^{-\alpha t}\cdot 1\cdot P\left(j|s,d(s)\right)dt}\right]}}{\lambda}_{s,d(s)}{e}^{-{\lambda}_{s,d(s)}u}du\hfill \\ {}\mathrm{f}\mathrm{o}\mathrm{r}\ \mathrm{s}\in \left\{1\right\},\ \mathrm{j}\in \mathrm{S},\ \mathrm{d}\left(\mathrm{s}\right)=\left\{1\right\},\hfill \end{array} $$

$$ \begin{array}{l}\overline{r}\left(s,d(s)\right)={\overline{\gamma}}_{\mathrm{nurse}}(s)+{\overline{\gamma}}_{\mathrm{RRT}}(s)\hfill \\ {}+{\displaystyle {\int}_0^{\infty}\left[{\displaystyle {\int}_0^u{e}^{-\alpha t}\cdot 1\cdot P\left(4f|s,d(s)\right)dt}\right]}{\lambda}_{s,d(s)}{e}^{-{\lambda}_{s,d(s)}u}du\hfill \\ {}\mathrm{f}\mathrm{o}\mathrm{r}\ \mathrm{s}\in \left\{1\right\},\ \mathrm{j}=\left\{4\mathrm{f}\right\},\ \mathrm{d}\left(\mathrm{s}\right)=\left\{2\right\},\hfill \end{array} $$

$$ \begin{array}{l}\overline{r}\left(s,d(s)\right)={\overline{\gamma}}_{\mathrm{nurse}}(s)\hfill \\ {}+{\displaystyle {\int}_0^1{\displaystyle {\sum}_{j\in \left\{1,2,3,6\right\}}P\left(j|4f,\ d(4f),Z<1\right)}\left[{\displaystyle {\int}_0^u{e}^{-\alpha t}\cdot 1\cdot d}t\right]{\lambda}_Z{e}^{-{\lambda}_Zu}du}\hfill \\ {} + {e}^{-{\lambda}_Z}{\displaystyle {\int}_0^1{e}^{-\alpha t}\cdot 1\cdot dt}\hfill \\ {}\mathrm{f}\mathrm{o}\mathrm{r}\ \mathrm{s}\in \left\{4\mathrm{f}\right\},\ \mathrm{d}\left(\mathrm{s}\right)\in \left\{1\right\}.\hfill \end{array} $$

where ${\overline{\gamma}}_{nurse}(s)= \max \left\{{\gamma}_{nurse}(s):s\in S\right\}$ and ${\overline{\gamma}}_{RRT}(s)= \max \left\{{\gamma}_{RRT}(s):s\in S\right\}$ as observed in the EMRs. We assume that a care provider who is more conservative regarding nurse and RRT resource needs in state *s* assigns a higher weight to $\overline{r}\left(s,d(s)\right)$, i.e., a lower value to *p*(*s*). For states corresponding to NEWS = 0 or discharge, we assume that providers do not assign a positive weight to $\overline{r}\left(s,d(s)\right)$, i.e., *p*(*s*) = 1 for *s* ∈ {4*f*, 4*s*, 5, 6}. This is supported by the assumption that for a stable, stabilized, or discharged patient, nurse- and RRT-related average time parameters derived from EMRs are considered a good approximation of the true patient needs according to clinical expert opinion. Derivation of the expressions of $\overline{r}\left(s,d(s)\right)$ are in Appendix 2, Eq. (2a–2e) and (3b–3f).

The term *ξ*(*s*) is a ratio representing the care provider’s perception of the workload associated with RRT time relative to nurse time for a patient in health states *s* ∈ *S*:

$$ \xi (s)=\frac{I\left(d(s)=2\right) \cdot {\mathrm{time}}_{\mathrm{RRT}}(s)}{\gamma_{\mathrm{nurse}}(s)} $$

The term time_RRT_(*s*) represents the actual RRT time observed from activation until departure from the patient’s bedside given the patient was in health state *s* at the time of RRT activation. In other words, the higher *ξ*(*s*), the more the provider values RRT-related resource time compared with the additional nurse time for stabilizing a patient in state *s*. Because RRT calls are not allowed in health states {4*f*, 4*s*, 5, 6}, *ξ*(*s*), takes the value zero in these states.

#### Modeling care provider heterogeneity

We define a care provider *profile* as a set of *weights* {*ρ*(*s*), *s* ∈ *S*} representing the care provider’s resource time-based perception of resource requirements and *ratios* {*ξ*(*s*), *s* ∈ *S*} representing the care provider’s valuation of RRT evaluation and assessment time relative to nurse time for a patient in a given health state *s*. The inverse transformation method is used to simulate 100 care provider profiles. The simulation refers to sampling time-perception measures *p*(*s*) and *ξ*(*s*) from probabilistic distributions. The parameter *p*(*s*) is generated from a Uniform [*a*(*s*)], *b*(*s*) distribution by selecting a state-dependent range [*a*(*s*), *b*(*s*)], determined by clinical expert opinion, from which the provider is equally likely to choose weights *p*(*s*) for s ∈ {1,2,3}. Based on clinical expert opinion, the range [*a*(*s*), *b*(*s*)] is greater in states 2 and 3 compared with the range in state 1. The health states 2 and 3 represent slightly concerning and concerning conditions due to the elevated NEWS; however, in many cases a patient’s condition can move in either direction (i.e., the patient can recover on their own, stabilize, or further deteriorate). This uncertainty is reflected by a wider range for the weights. Further, a Weibull [*θ*(*s*), *φ*(*s*)] distribution with state-dependent shape parameter *θ*(*s*) and scale parameter *φ*(*s*) is used to generate *ξ*(*s*). The Weibull distribution is identified as a good fit for the simulated data from (self-identifying name of the facility omitted). The input parameters for the distributions are summarized in Table 2.

**Table 2:** Distribution of time-perception measures for the modified SMDP model

NEWS	Health state	Distribution of *ρ* ($s$)	Distribution of $\xi$($s$)
0 or discharge	{4f, 4s, 5, 6}	1	0
1–4	3	UNIF (0.2, 0.6)	WEIBULL (1.04, 0.414)
5−6 or individual physiological parameter with significant abnormality	2	UNIF (0, 0.4)	WEIBULL (0.587, 0.879)
≥7	1	UNIF (0, 0.2)	WEIBULL (0.734, 0.855)

Once the input parameters are identified, we generated sets of *p*(*s*) and *ξ*(*s*) for s ∈ S {1,2,3} to represent care provider profiles which are divided into groups using cluster analysis.

#### Classifying care providers

Cluster analysis is a method for partitioning data into groups of objects such that objects in the same cluster are more similar to each other than to objects residing in other clusters [36]. In this study, the objects are the provider profiles defined as a set of random variables [*p*(*s*), *ξ*(*s*)] with s ∈ S. A cluster is a homogenous group of provider profiles with similar resource time perceptions. The clusters are identified with hierarchical clustering algorithms using the distance between objects to identify the clusters. A modified SMDP model is developed for each cluster using the corresponding cluster average values for *p*(*s*) and ξ(*s*). Three Baseline Scenarios and five Modified Scenarios were analyzed for the cluster analyses (Table 3).

**Table 3:** Clustering variables, algorithms for the baseline and modified scenarios

Scenario	Clustering variables	Clustering algorithm
Baseline #1	$$ \begin{array}{c}\hfill \rho (3),\ \rho (2),\rho (1)\hfill \\ {}\hfill \xi (3),\xi (2),\xi (1)\hfill \end{array} $$	Single Linkage
Baseline #2		Ward’s Method
Baseline #3		Centroid
Modified #1		Single Linkage (with increased Uniform upper bounds)
Modified #2		Single Linkage (with decreased Uniform upper bounds)
Modified #3		Single Linkage (with increased Uniform upper and lower bounds)
Modified #4	*ξ*(3), *ξ*(2), *ξ*(1)	Single Linkage
Modified #5	*ρ*(3), *ρ*(2), *ρ*(1)	Single Linkage

The Baseline Scenarios explore how the cluster structure and content differ depending on the clustering algorithm. Modified Scenarios were developed by changing Baseline Scenario 1 by: (i) using wider or narrower bounds on the Uniform distribution for *p*(*s*) for *s* ∈ {1,2,3} (Modified Scenarios 1–3); and (ii) using a subset of the clustering variables (Modified Scenarios 4–5).

#### Modified SMDP model formulation

The modified SMDP model is solved using the primal and dual Linear Programs (LP). As a numerical example, we present the results for medical patients with moderate ROD, which can be easily applied to other subpopulations. Let {*β*(*s*), *s* ∈ *S*} denote arbitrarily selected positive scalars satisfying ∑_*s* ∈ *S*_ *β*(*s*) = 1, which allows the interpretation of this set as a probability distribution on the state space *S*. The associated dual LP identifies the optimal policy for each state, while the primal LP calculates the total expected discounted stabilization and resource time for each state [20]. The dual LP formulation is

$$ \mathrm{Minimize}\ {\displaystyle {\sum}_{s\ \in\ S}{\displaystyle {\sum}_{d\ \in A}{r}^{\hbox{'}}\left(s,d\right)x\left(s,d\right)}} $$

subject to∑_*d* ∈ *A*_ *x*(*j*, *d*) − ∑_*s* ∈ *S*_∑_*d* ∈ *A*_ [∫_0_^∞^ *e* ^− *αt*^ *P*(*j*|*s*, *d*)*F*(*dt*|*s*, *d*)] *x*(*s*, *d*) = *β*(*s*) for all *j* ∈ *S x*(*s*, *d*) ≥ 0  for *s* ∈ *S*, *d* ∈ *A*.

The term *x*(*s*, *d*) represents the total discounted joint probability that the patient is in state *s* and the provider chooses *d* under the distribution {*β*(*j*)} [20]. If {*x*(*s*, *d*) : *s* ∈ *S*, *d* ∈ *A*} is a feasible solution to the dual LP, then the stationary policy is given by

$$ P\left\{d(s)=a\right\}=\frac{x\left(s,a\right)}{{\displaystyle {\sum}_{d\in A}}x\left(s,d\right)}\;\mathrm{f}\mathrm{o}\mathrm{r}\;a\in \mathrm{A} $$

where *P*{*d*(*s*) = *a*} is the probability of selecting the action *a* in state *s*. The primal LP formulation is

$$ \mathrm{Maximize}{\displaystyle {\sum}_{s\in S}\beta (s){v}_{\alpha, \pi }(s)} $$

subject to*v*_*α*,*π*_(*s*) − ∑_*j* ∈ *S*_[∫_0_^∞^ *e* ^− *αt*^ *P*(*j*|*s*, *d*)*F*(*dt*|*s*, *d*)] *v*_*α*,*π*_(*j*) ≤ *r* ^'^(*s*, *d*) for *d* ∈ *A* and *s* ∈ *S v*_*α*,*π*_(*s*) unconstrained for *s* ∈ *S*.

The primal and dual LP formulations are solved for each Baseline and Modified Scenario and each cluster for the selected subpopulation. For a cluster containing one profile, the corresponding time perception measures, *p*(*s*) and, *ξ*(*s*) are used in the LP models. If a cluster contains multiple profiles, we compute cluster averages of *p*(*s*) and *ξ*(*s*), and solve the LP models using these average values.

## Results

### Case study setting

This is a retrospective study using patient-level data extracted from the EMRs provided by (self-identifying name of the facility omitted). The study cohort includes 55,385 adult general ward patients hospitalized at the facility from January 2011 to December 2012. The inclusion criteria are age at admission (≥18 years) and care location (only data collected during a stay in the general ward are used).

### Baseline SMDP model results

For each subpopulation, the optimal total stabilization-related time and the optimal RRT-activation policy were computed using the Policy Iteration Algorithm [20]. State transition probabilities and sojourn time distributions were derived from the EMRs using Maximum Likelihood Estimates (MLEs) [37]. State-dependent time parameters were derived from EMRs. Empirical analysis suggested that the exponential distribution is a good fit for the sojourn time distribution for all model states, except state 4*f* where the sojourn time is restricted to be in [0,1] by the definition of stabilization. Sojourn time distribution in state 4*f* is presented in the Appendices 2 and 3. Table 4 shows the optimal NEWS-based RRT-activation thresholds by subpopulation.

**Table 4:** Optimal policy by subpopulation where A: medical with low ROD; B: surgical with low ROD; C: medical with moderate ROD; D: surgical with moderate ROD; E: medical with high ROD; and F: surgical with high ROD

Subpopulation	Optimal policy			
	NEWS 0 “Stable”	NEWS 1–4 “Slightly concerning”	NEWS 5–6 or individual physiological parameter with significant abnormality “Concerning”	NEWS ≥ 7 “Distress”
B	Wait		Patient may benefit from activating RRT	
A, C–E	Wait	Patient may benefit from activating RRT		

Optimal policies suggest RRT activation for NEWS values exceeding a critical threshold which differs by subpopulation. Activating RRT immediately is optimal for patients with a “slightly concerning” or worse health state (NEWS values above 0) for all subpopulations, except surgical patients with low ROD (denoted by B) for whom it is optimal to wait until the patient is in a “concerning” health state (i.e., threshold NEWS >4). This result suggests there are two critical patient populations: surgical patient with low ROD and all other patients, with different RRT activation criteria. This translates to a simple rule for activating RRT with two thresholds, NEWS >4 for subpopulation B, and NEWS >0 for the other considered subpopulations. These results imply that surgical patients with low ROD may be healthier and better able to recover from deterioration on their own. From a clinical perspective, surgical patients can be hospitalized for a non-urgent elective surgery whereas medical patients are admitted due to discomfort and possibly symptoms of acute or chronic conditions. Thus, surgical patients with low ROD may recover from a NEWS value below 4 without additional RRT intervention, whereas medical patients with low ROD benefit from an RRT intervention at a lower NEWS threshold. Further, if activating RRT is the optimal action for a given health state, then it is the optimal action for all worse health states, i.e., there is a state-dependent control-limit structure. This result categorizes RRT activation rules into two sets of policies, and simplifies clinical implementation.

For all subpopulations A–E, total expected stabilization and resource time increases as the patient’s condition deteriorates. Further, the total expected stabilization and resource times in health states {4*f*, 4*s*, 5} are similar for some subpopulations (A, C, and D) and differ for others (B, E, and F). Specifically, the total expected cost for high ROD patients depends on the stabilization condition, i.e., if a patient’s NEWS has not exceeded 0 (i.e., never reached the “slightly concerning” level) and the patient is in health state 5, or the patient’s NEWS exceeded 0 (has reached or exceeded the “slightly concerning” level), which resulted in moving to health states 4*f* or 4*s*. This difference may occur because high ROD patients may not present in health state 5 at the beginning of a hospitalization episode due to their physiological condition, or they may not remain in this health state, which impacts the resource time accumulated in state 5. In addition, surgical patients are more resource intensive (in terms of time required for their care) than medical patients for any given ROD category. Surgical patients may stay longer in the general ward before and after the procedure, which may result in higher resource times, and possible TTS and FTR. In addition, surgical patients may require more intense care even when they are in better health states.

With the goal of demonstrating that the proposed model brings benefits to a clinical setting, we used a time-based metric focusing on patients who experienced an RRT activation during the study period: average time to activate RRT, defined as the average time from the beginning of a hospitalization episode until RRT activation criteria are met. Specifically, for a selected subpopulation we compared the average time to activate RRT using the current policy (as indicated by the observed RRT activation times in the dataset) with the hypothetical average time to activate RRT if the proposed model was used during the same time period. The comparison was applied to one subpopulation over the 2-year time period (January 2011–December 2012), which can be easily applied to other subpopulations. Medical patients with moderate ROD was selected for comparison because they represent the largest subpopulation with a high RRT incidence (18,803 unique patients, mean NEWS 1.99, standard deviation of NEWS 2.14, minimum NEWS 0, maximum NEWS 17, and 827 RRT activations). Compared to the current policy, the optimal RRT policy resulted on average in 5.2 h earlier RRT activation. Based on clinical expert opinion and relevant literature, serious undesired events followed by APD expose patients to an increased risk of death [38]. Many of these events result from insufficient or delayed medical care [38]. For example, it has been shown that majority of cardiopulmonary arrests are preceded by dramatic changes in vital signs or other clinical decline during a 6–8 h period before the arrest [6]. Earlier recognition of the need for RRT activation by the proposed model can improve the stabilization of patients, and potentially reduce the incidence of undesired outcomes, including death.

Overall, results from this study highlight the importance of a personalized approach when treating APD, and provide optimal subpopulation-specific RRT-activation rules with insight into stabilization and resource time requirements.

### Modified SMDP results

The clustering analysis was applied to subpopulation C (medical patients with moderate ROD) as an example, which can be easily expanded to other subpopulations. The results showed that different clustering algorithms impact the size and content of clusters. All scenarios identified a main cluster and multiple “outlier” clusters consisting of one profile. The main cluster was characterized by increasing time-perception measure *p*(*s*) as the health condition worsens, i.e., a provider in the main cluster assigned a higher weight to the maximum nurse and RRT times, assuming the physiological deterioration in these states will require high RRT and nurse resource use. The outlier clusters tended to have higher valuation of the RRT time relative to the nurse time compared with the main cluster. Baseline Scenario 1 (Single Linkage Method) identified four clusters, including one main cluster and three outlier clusters (Appendix 5, Table 20). Baseline Scenario 2 (Ward’s Method) and Baseline Scenario 3 (Centroid Method) identified the same main cluster and outliers as in Baseline Scenario 1, and additional outlier profiles were separated from the main cluster (Appendix 5, Tables 21 and 22). Further, the Modified Scenarios showed that changing the Uniform distribution input parameters, such as increasing the upper bound or decreasing the lower bound, further partitioned the main cluster and separated additional outliers from the main cluster (Appendix 5, Tables 23, 24, 25, 26 and 27).

Table 5 presents the LP results including the total expected stabilization and resource time in hours and optimal RRT activation policy for the Baseline Scenario 1 in comparison with the baseline SMDP model results for medical patients with moderate ROD.

**Table 5:** LP Results for baseline and modified SMDP models for medical patients with moderate ROD

	Optimal value function [hrs.]						Optimal policy d*(s)					
	Baseline SMDP model results											
	v(5)	v(4s)	v(4f)	v(3)	v(2)	v(1)	d*(5)	d*(4s)	d*(4f)	d*(3)	d*(2)	d*(1)
	6.66	7.30	9.46	10.69	12.38	12.79	Wait			Call		
	Baseline scenario #1 using single linkage											
Cluster #	v(5)	v(4s)	v(4f)	v(3)	v(2)	v(1)	d*(5)	d*(4s)	d*(4f)	d*(3)	d*(2)	d*(1)
1	10.22	11.37	11.47	12.50	15.66	15.67	Wait			Call		
2	10.59	11.05	11.16	11.88	14.57	16.24						Wait
3	10.55	10.97	11.08	12.22	15.14	15.79						
4	11.20	12.42	12.50	13.90	15.26	15.53						

Table 5 shows that for all clusters under the Baseline Scenario 1, the total expected stabilization-related times are non-increasing in state $s$. Further, the total expected stabilization and resource time for each state is higher in the modified SMDP model compared with the baseline SMDP (Appendix 5, Table 19). In the modified SMDP model under all scenarios, Cluster 2 includes the same outlier profile characterized by a high perception of RRT time relative to nurse time in clinical distress state (i.e., state 1). For this cluster, the optimal policy in state 1 is to wait (i.e., not use the RRT resources and the decision about the patient’s care is made by the bedside provider) rather than activating RRT immediately (Appendix 5, Tables 20, 21, 22, 23, 24, 25, 26 and 27). In clinical practice, activating RRT is a decision made by the bedside provider. Based on clinical expert opinion, if a bedside provider has a high valuation of RRT time compared with nurse time, he/she may consider activating RRT in slightly concerning and concerning states (states 2 and 3) to obtain additional critical care guidance because of the uncertainty associated with the patient’s clinical path. However, in clinical distress state (state 1), the bedside provider may prefer waiting to avoid wasting any critical care resources because the decision about the patient’s care is clear due to severity of the physiological condition. This is different than the baseline SMDP optimal policy in health state 1, which is to call RRT immediately. This result highlights the impact of a provider’s perception on the optimal RRT activation policy. The remainder of the LP results is presented in Appendix 5.

## Discussion

As opposed to focusing on disease-specific interventions, APD requires a system-wide approach with an emphasis on patient characteristics because it can affect patients across diseases. Further, an acute-care delivery team may consist of providers with different time perceptions of resource requirements and estimates of patient needs. Thus, the recognition of, and response to, APD, e.g., RRT activation, may be affected by patient and provider heterogeneity. Measurement of RRT effectiveness and management in clinical practice remains a challenge. Although several studies focused on patient outcomes or team-based qualitative metrics, their findings highlight the need for enhanced metrics to assess the impact of RRT on patients’ clinical trajectories and care processes [12–15].

We hypothesized that the heterogeneity and uncertainty in acute-care systems may result in different RRT activation policies and further there are provider clusters, defined by providers’ perception of time requirements and relative valuation of stabilization-related resource needs, which differ in their RRT-activation policies. The baseline SMDP model identified subpopulation-specific RRT-activation thresholds and established a framework for incorporating patient heterogeneity in acute-care delivery. The modified SMDP model incorporated provider heterogeneity through variation in the value of time associated with stabilization. Results showed that the stabilization and resource time was higher in the modified model compared with the baseline model for all health states. This increase may be explained by the weighted average formulation for the stabilization and resource time functions, which allows the values of the nurse- and RRT-related time parameters to vary by provider, and by sampling the ratios *ξ*(*s*) from Weibull distributions, which allows the relative RRT time values to take larger values than those estimated by observed data. The LP results highlighted the impact of the selection of the clustering algorithm and the distributions for time-perception measures on RRT-activation policies. The optimal RRT policy was different for one outlier cluster with a high *ξ*(*s*) for which waiting (instead of activating RRT immediately) was optimal in the clinical distress state (state 1). A care provider in this profile might argue that for a patient in a clinical distress state (state 1), acute-care requirements might be clear due to the severity of the physiological condition, and therefore the provider may feel comfortable with making care decisions independent of RRT. Therefore, the increase in resource time due to RRT activation is not considered optimal in this health state.

By sampling time-perception measures from probablistic distributions and clustering provider profiles, the modified SMDP model identified cluster-specific optimal RRT policies. The results suggested that differences in stabilization and resource time perceptions may impact the resuscitation preferences of different providers in the clinically distressed state. This finding supports our hypothesis that the provider clusters differ in their RRT-activation preferences as well as their estimation of stabilization-related resource needs.

Informing RRT activation decisions and capturing the underlying stochastic deterioration process using a quantified score offers several opportunities to improve patient care. RRT is a scarce and valuable resource. Optimizing the RRT activation may decrease wasted time and resources due to the RRT activation for the wrong patient at the wrong time. In addition, in clinical practice, RRTs can occur simultaneously. Developing a patient-centered approach to RRT activation can help prioritize RRT calls.

Our study is motivated by the challenge of addressing the fact that patients were dying because providers have been failing to recognize acute changes in their clinical status. Traditional educational interventions were failing. A much more strategic, systems engineering and analytic approach was required. This study contributes to recognition and response to APD by analyzing key components of physiological deterioration and performing statistical analyses to identify patient subpopulations using clinical parameters, such as patients’ admission type and risk of deterioration. Using clustering methods, we identified distinct provider clusters with regard to their RRT activation preferences and estimation of resource needs to address provider heterogeneity in critical care settings. This work provided quantitative evidence to support the clinical research that “one-size-fits-all” criteria for alerting critical care teams in response to patient deterioration has not been effective and needs to be personalized to fit the needs of individual patients.

We acknowledge that there is some subjectivity in the current RRT activation criteria at the study hospital, e.g., the criteria regarding “concern about the patient”, and the optimal RRT rules derived from the SMDP models do not incorporate these subjective elements. In clinical practice, subjective clinical judgement of frontline providers contains valuable information that may not be captured by objective criteria. This is particularly critical for nursing assessment and the concern about a patient. Nursing assessment provides information necessary to support identification of problems and symptoms that are relevant for patient care [39]. In addition to vital signs and laboratory tests, nursing assessment provides better understanding of the patients’ physiological condition. In this context, subjectivity in the current RRT system is important in detecting signals of APD. While our SMDP models provide simple optimal RRT activation rules as clinical decision guidance, they do not aim to replace the subjective clinical judgement.

Future research could expand our work in several ways. A limitation of the current study is that the absorbing state represents all types of discharge from the general ward (i.e., transfer to higher-level care, discharge alive, discharge to hospice, or death). Future work could modify the terminal stabilization and resource time associated with different absorbing states based on discharge dispositions. Further, we recognize that different health systems may operate differently, and the results drawn from our study based on data from one hospital may not be generalizable. Therefore, future work would be to expand this framework for various facilities to study the dynamics of their specific patient subpopulations. Another limitation is that the relative RRT time perception measures *ξ*(1), *ξ*(2), *ξ*(3), were generated independently of the time perception measures *ρ*(1), *ρ*(2), *ρ*(3), and then combined to generate the simulated provider profiles. However, these measures may be dependent. For example, a provider who assigns a higher weight to the worse-case stabilization and resource time function in state *s* may select a higher relative RRT measure in this state, i.e., *ρ*(*s*) and *ξ*(*s*) may be correlated. An area for future research could explore the dependency structure between time perception measures by generating dependent random variables.

In conclusion, APD during hospitalization, while commonly signaled by abnormal vital signs hours before critical events, can be difficult to discern. EWS-based dynamic and stochastic models can aid data-driven clinical decision making by enhancing the ability to capture changes in patient condition over time in a patient-centered manner. This is particularly applicable for providers in an environment where patients are monitored at irregular intervals, e.g., the general ward. Using EWS-based approaches, standardized and structured communication between the provider team members can help to mitigate communication errors arising from fragmented nature of health care delivery and frequent handoffs.

The methods described in this study extend the existing literature by analyzing the stochastic nature of APD, evaluating health-state dependent resource needs, and incorporating both patient and provider heterogeneity in a unique framework. While our model is developed for adult patients, our approach can be easily extended to different patient subpopulations. The identified optimal RRT policies utilize physiological measures that are readily available during routine hospital rounding. In addition, the modified SMDP model provides insight regarding stabilization and resource time as a function of the patient type and the providers’ estimates of stabilization-related patient needs. These methods can be used by hospitals to guide RRT-activation policies in clinical practice for patients who present with APD or develop APD during a hospitalization. Moreover, these methods provide a better understanding for the impact of provider heterogenity on resuscitation actions and time-based stabilization-related resource use.

## APPENDIX 1: Transition rate matrices (TRM)

In the following 7 × 7 transition rate matrices, rows represent current state *s* and columns represent next state *j* the patient moves after leaving the current state. The values in the cells represent transition rates in transitions/h given the current health state *s* (denoted by the row), the decision maker chooses action *d*(*s*), and the next state transition is to state *j* (denoted by the column) as derived from EMRs provided by (self-identifying name of the facility omitted) used in our case study.

**Table 6:** Transition rate matrix for medical patients with low ROD, and for the action wait

	5	4S	4F	3	2	1	6
5	−0.4726	0.0000	0.0000	0.1246	0.2871	0.0000	0.0610
4S	0.0000	−0.6639	0.0000	0.2183	0.1803	0.1749	0.0904
4F	0.0000	0.3677	−1.0316	0.2183	0.1803	0.1749	0.0904
3	0.0000	0.0000	0.1563	−0.7708	0.2503	0.2925	0.0717
2	0.0000	0.0000	0.3167	0.8328	−2.3316	0.8961	0.2860
1	0.0000	0.0000	0.3030	0.8758	1.1247	−2.6919	0.3883
6	0.0000	0.0000	0.0000	0.0000	0.0000	0.0000	1.0000

**Table 7:** Transition rate matrix for medical patients with low ROD, and for the action activate RRT

	5	4S	4F	3	2	1	6
5	−0.4726	0.0000	0.0000	0.1246	0.2871	0.0000	0.0610
4S	0.0000	−0.6639	0.0000	0.2183	0.1803	0.1749	0.0904
4F	0.0000	0.3677	−1.0316	0.2183	0.1803	0.1749	0.0904
3	0.0000	0.0000	0.1100	−0.2300	0.0000	0.0000	0.1200
2	0.0000	0.0000	0.89	0.0000	−1.3900	0.0000	0.5000
1	0.0000	0.0000	0.56	0.0000	0.0000	−0.8200	0.2600
6	0.0000	0.0000	0.0000	0.0000	0.0000	0.0000	1.0000

**Table 8:** Transition rate matrix for surgical patients with low ROD, for the action wait

	5	4S	4F	3	2	1	6
5	−2.1234	0.0000	0.0000	0.2152	0.3211	1.0000	0.5871
4S	0.0000	−3.3571	0.0000	0.2549	0.3312	2.6667	0.1043
4F	0.0000	0.1083	−3.4654	0.2549	0.3312	2.6667	0.1043
3	0.0000	0.0000	0.1909	−1.2145	0.4029	0.4994	0.1212
2	0.0000	0.0000	0.4248	1.0750	−3.3357	1.3449	0.4911
1	0.0000	0.0000	4.6154	1.0611	1.2981	−8.2789	1.3043
6	0.0000	0.0000	0.0000	0.0000	0.0000	0.0000	1.0000

**Table 9:** Transition rate matrix for surgical patients with low ROD, and for the action activate RRT

	5	4S	4F	3	2	1	6
5	−2.1234	0.0000	0.0000	0.2152	0.3211	1.0000	0.5871
4S	0.0000	−3.3571	0.0000	0.2549	0.3312	2.6667	0.1043
4F	0.0000	0.1083	0.1909	−1.2145	0.4029	0.4994	0.1212
3	0.0000	0.0000	1.3300	−1.4500	0.0000	0.0000	0.1200
2	0.0000	0.0000	0.8900	0.0000	−1.3900	0.0000	0.5000
1	0.0000	0.0000	0.5600	0.0000	0.0000	−0.8200	0.2600
6	0.0000	0.0000	0.0000	0.0000	0.0000	0.0000	1.0000

**Table 10:** Transition rate matrix for medical patients with moderate ROD, and for the action wait

	5	4S	4F	3	2	1	6
5	−0.4838	0.0000	0.0000	0.1020	0.1341	0.1923	0.0554
4S	0.0000	−0.6700	0.0000	0.1799	0.2298	0.1703	0.0900
4F	0.0000	0.3676	−1.0377	0.1799	0.2298	0.1703	0.0900
3	0.0000	0.0000	0.1191	−0.6207	0.2173	0.2269	0.0574
2	0.0000	0.0000	0.3666	0.5871	−1.8825	0.6806	0.2482
1	0.0000	0.0000	0.5093	0.5131	0.6221	−1.8700	0.2255
6	0.0000	0.0000	0.0000	0.0000	0.0000	0.0000	1.0000

**Table 11:** Transition rate matrix for medical patients with moderate ROD, and for the action activate RRT

	5	4S	4F	3	2	1	6
5	−0.4838	0.0000	0.0000	0.1020	0.1341	0.1923	0.0554
4S	0.0000	−0.6700	0.0000	0.1799	0.2298	0.1703	0.0900
4F	0.0000	0.3676	−1.0377	0.1799	0.2298	0.1703	0.0900
3	0.0000	0.0000	0.1000	−0.2000	0.0000	0.0000	0.1000
2	0.0000	0.0000	0.96	0.0000	−1.5200	0.0000	0.5500
1	0.0000	0.0000	0.48	0.0000	0.0000	−0.7700	0.2900
6	0.0000	0.0000	0.0000	0.0000	0.0000	0.0000	1.0000

**Table 12:** Transition rate matrix for surgical patients with moderate ROD, and for the action wait

	5	4S	4F	3	2	1	6
5	−2.9777	0.0000	0.0000	0.2052	0.3295	2.3077	0.1353
4S	0.0000	−0.7826	0.0000	0.2227	0.2631	0.2082	0.0886
4F	0.0000	0.3643	−1.1469	0.2227	0.2631	0.2082	0.0886
3	0.0000	0.0000	0.1264	−0.7632	0.2666	0.3139	0.0563
2	0.0000	0.0000	0.3953	0.7859	−2.4371	0.9311	0.3247
1	0.0000	0.0000	0.4438	0.8763	0.9212	−2.6171	0.3758
6	0.0000	0.0000	0.0000	0.0000	0.0000	0.0000	1.0000

**Table 13:** Transition rate matrix for surgical patients with moderate ROD, and for the action activate RRT

	5	4S	4F	3	2	1	6
5	−2.9777	0.0000	0.0000	0.2052	0.3295	2.3077	0.1353
4S	0.0000	−0.7826	0.0000	0.2227	0.2631	0.2082	0.0886
4F	0.0000	0.3643	−1.1469	0.2227	0.2631	0.2082	0.0886
3	0.0000	0.0000	0.6600	−0.94	0.0000	0.0000	0.2800
2	0.0000	0.0000	4.1400	0.0000	−5.02	0.0000	0.8800
1	0.0000	0.0000	0.8400	0.0000	0.0000	−1.0300	0.1800
6	0.0000	0.0000	0.0000	0.0000	0.0000	0.0000	1.0000

**Table 14:** Transition rate matrix for medical patients with high ROD, and for the action wait

	5	4S	4F	3	2	1	6
5	−1.8293	0.0000	0.0000	0.1881	0.1412	0.0000	1.5000
4S	0.0000	−0.7132	0.0000	0.2015	0.2385	0.1598	0.1133
4F	0.0000	0.3668	−1.0799	0.2015	0.2385	0.1598	0.1133
3	0.0000	0.0000	0.1171	−0.6395	0.2291	0.2391	0.0542
2	0.0000	0.0000	0.3076	0.5043	−1.7019	0.6201	0.2698
1	0.0000	0.0000	0.1716	0.4354	0.5126	−1.2421	0.1226
6	0.0000	0.0000	0.0000	0.0000	0.0000	0.0000	1.0000

**Table 15:** Transition rate matrix for medical patients with high ROD, and for the action activate RRT

	5	4S	4F	3	2	1	6
5	−1.8293	0.0000	0.0000	0.1881	0.1412	0.0000	1.5000
4S	0.0000	−0.7132	0.0000	0.2015	0.2385	0.1598	0.1133
4F	0.0000	0.3668	−1.0799	0.2015	0.2385	0.1598	0.1133
3	0.0000	0.0000	0.0300	−0.9600	0.0000	0.0000	0.9300
2	0.0000	0.0000	0.5000	0.0000	−0.6500	0.0000	0.1500
1	0.0000	0.0000	0.8800	0.0000	0.0000	−1.1500	0.2700
6	0.0000	0.0000	0.0000	0.0000	0.0000	0.0000	1.0000

**Table 16:** Transition rate matrix for surgical patients with high ROD, and for the action wait

	5	4S	4F	3	2	1	6
5	−1.7679	0.0000	0.0000	0.3896	0.0740	0.0000	1.3043
4S	0.0000	−1.1958	0.0000	0.3629	0.4395	0.2703	0.1232
4F	0.0000	0.3316	−1.5274	0.3629	0.4395	0.2703	0.1232
3	0.0000	0.0000	0.1561	−0.8138	0.3215	0.2718	0.0642
2	0.0000	0.0000	0.4456	0.8096	−2.5917	0.8190	0.5175
1	0.0000	0.0000	0.6218	0.6520	0.6534	−2.1606	0.2335
6	0.0000	0.0000	0.0000	0.0000	0.0000	0.0000	1.0000

**Table 17:** Transition rate matrix for surgical patients with high ROD, and for the action activate RRT

	5	4S	4F	3	2	1	6
5	−1.7679	0.0000	0.0000	0.3896	0.0740	0.0000	1.3043
4S	0.0000	−1.1958	0.0000	0.3629	0.4395	0.2703	0.1232
4F	0.0000	0.3316	−1.5274	0.3629	0.4395	0.2703	0.1232
3	0.0000	0.0000	1.4000	−2.3300	0.0000	0.0000	0.9300
2	0.0000	0.0000	2.4000	0.0000	−2.5500	0.0000	0.1500
1	0.0000	0.0000	1.3300	0.0000	0.0000	−1.4400	0.1100
6	0.0000	0.0000	0.0000	0.0000	0.0000	0.0000	1.0000

## APPENDIX 2: Stabilization and resource time functions in the SMDP models

We define the term *λ*_*s*,*d*(*s*)_ as the state- and action-dependent exponential rate parameter for the sojourn time distribution in state *s* until the next decision epoch. For states *s* ∈ *S*′, where *S*′ = *S*\{4*f*, 6}, the sojourn time corresponds to the minimum of competing exponential delays. Therefore, for the states *s* ∈ *S*’, the sojourn time in health state *s* follows an exponential distribution with rate parameter *λ*_*s*,*d*(*s*)_ = ∑_*j* ∈ *S*, *j* ≠ *s*_ *λ*_*s*,*d*(*s*),*j*_ where *λ*_*s*,*d*(*s*),*j*_ is the rate corresponding to the exponential time spent in *s* before a transition to *j* would occur, given action *d*(*s*); and corresponding to impossible state transitions, we make the following assignment of zero rates:

0a $$ {\lambda}_{s,d(s),j}=0\;\mathrm{f}\mathrm{o}\mathrm{r}\;s\in {S}^{\hbox{'}}\;\mathrm{and}\;j\in \left\{4s,\ 5\right\}, $$

0b $$ {\lambda}_{s,d(s),s}=0\;\mathrm{f}\mathrm{o}\mathrm{r}\;s\in \left\{1,\ 2,\ 3\right\}, $$

and

0c $$ {\lambda}_{s,d(s),4f}=0\;\mathrm{f}\mathrm{o}\mathrm{r}\;s\in \left\{4s,\ 5\right\}. $$

With Eq. (0a–c) as well as the usual rate assignment of *λ*_*s*,*d*(*s*),*j*_ for each *s* ∈ *S*′ and for each *j* ∈ *S* not covered by (0a–c), the sojourn time in state *s* is exponentially distributed with a mean of 1/*λ*_*s*,*d*(*s*)_ hours.

For state 4*f*, the sojourn time conditional on the action d(4*f*), is denoted by *H*_4*f*,*d*(4*f*)_, is concentrated in [0, 1], and is formulated as follows:

1a $$ P\left({H}_{4f,d(4f)}=1\right)=P\left({ \min}_{j\in \left\{1,2,3,6\right\}}{H}_{4f,d(4f),j}>1\right)={e}^{-{\displaystyle {\sum}_{j\in \left\{1,2,3,6\right\}}{\lambda}_{4f,d(4f),j}}}, $$

where for *j* ∈ {1, 2, 3, 6}, the independent random variable *H*_4*f*,*d*(4*f*),*j*_ representing the potential time to make the transition 4*f* → *j* is exponentially distributed with rate parameter *λ*_4*f*,*d*(4*f*),*j*_. Therefore, we have

1b $$ P\left(0<{H}_{4f,d(4f)}<1\right)=1-P\left({H}_{4f,d(4f)}=1\right)=1-{e}^{-{\displaystyle {\sum}_{j\in \left\{1,2,3,6\right\}}{\lambda}_{4f,d(4f),j}}} $$

so that the cumulative distribution function of *H*_4*f*,*d*(4*f*)_ is given by

1c $$ P\left({H}_{4f,d(4f)}\le u\right)=\left\{\begin{array}{cc}\hfill \begin{array}{c}\hfill 1-{e}^{-u{\displaystyle {\sum}_{j\in \left\{1,2,3,6\right\}}{\lambda}_{4f,d(4f),j}\ }\ }\ \hfill \\ {}\hfill \kern9.74em \hfill \end{array}\hfill & \hfill \mathrm{if}\ 0\le u<1\hfill \\ {}\hfill 1\hfill & \hfill \mathrm{if}\ u\ge 1\hfill \end{array}\right., $$

The function *r*(*s*, *d*(*s*)) is given by:

2a $$ r\left(s,d(s)\right)=0\kern0.75em \mathrm{f}\mathrm{o}\mathrm{r}\ \mathrm{s}\in \left\{5,\ 4\mathrm{s}\right\},\mathrm{d}\left(\mathrm{s}\right)=\left\{1\right\}, $$

2b $$ \begin{array}{c}\hfill r\left(s,d(s)\right) = {\gamma}_{\mathrm{nurse}}(s)+I\left(d(s)=2\right)\ {\gamma}_{\mathrm{RRT}}(s)\hfill \\ {}\hfill +{\displaystyle {\int}_0^{\infty}\left[{\displaystyle {\int}_0^u{e}^{-\alpha t}c\left(s,4f\right)P\left(4f|s,d(s)\right)dt}\right]}{\lambda}_{s,d(s)}{e}^{-{\lambda}_{s,d(s)}u}du\hfill \\ {}\hfill \mathrm{f}\mathrm{o}\mathrm{r}\ \mathrm{s}\in \left\{2,\ 3\right\},\ \mathrm{j}=\left\{4\mathrm{f}\right\},\ \mathrm{d}\left(\mathrm{s}\right)\in \left\{1,\ 2\right\},\hfill \end{array} $$

2c $$ \begin{array}{l}r\left(s,d(s)\right) = {\gamma}_{\mathrm{nurse}}(s)\hfill \\ {}+{\displaystyle {\int}_0^{\infty}\left[{\displaystyle {\int}_0^u{e}^{-\alpha t}c\left(s,4f\right)P\left(4f|s,d(s)\right)dt}\right]}{\lambda}_{s,d(s)}{e}^{-{\lambda}_{s,d(s)}u}du\hfill \\ {}+{\displaystyle {\int}_1^{\infty }{\displaystyle {\sum}_{j\in S}\left[{\displaystyle {\int}_0^u{e}^{-\alpha t}c\left(s,j\right)P\left(j|s,d(s)\right)dt}\right]}}{\lambda}_{s,d(s)}{e}^{-{\lambda}_{s,d(s)}u}du\hfill \\ {}\mathrm{f}\mathrm{o}\mathrm{r}\ \mathrm{s}\in \left\{1\right\},\ \mathrm{j}\in \mathrm{S},\ \mathrm{d}\left(\mathrm{s}\right)=\left\{1\right\},\hfill \end{array} $$

2d $$ \begin{array}{l}r\left(s,d(s)\right) = {\gamma}_{\mathrm{nurse}}(s)+{\gamma}_{\mathrm{RRT}}(s)\hfill \\ {}+{\displaystyle {\int}_0^{\infty}\left[{\displaystyle {\int}_0^u{e}^{-\alpha t}c\left(s,4f\right)P\left(4f|s,d(s)\right)dt}\right]}{\lambda}_{s,d(s)}{e}^{-{\lambda}_{s,d(s)}u}du\hfill \\ {}\mathrm{f}\mathrm{o}\mathrm{r}\ \mathrm{s}\in \left\{1\right\},\ \mathrm{j}=\left\{4\mathrm{f}\right\},\ \mathrm{d}\left(\mathrm{s}\right)=\left\{2\right\},\hfill \end{array} $$

2e $$ \begin{array}{l}r\left(s,d(s)\right) = {\gamma}_{\mathrm{nurse}}(s)\hfill \\ {}+{\displaystyle {\int}_0^1{\displaystyle {\sum}_{j\in \left\{1,2,3,6\right\}}P\left(j|4f,\ d(4f),Z<1\right)}}\left[{\displaystyle {\int}_0^u{e}^{-\alpha t}c\left(4f,j\right)dt}\right]{\lambda}_Z{e}^{-{\lambda}_Zu}du\hfill \\ {} + {e}^{-{\lambda}_Z}{\displaystyle {\int}_0^1{e}^{-\alpha t}c\left(4f,4s\right)dt}\hfill \\ {}\mathrm{f}\mathrm{o}\mathrm{r}\ \mathrm{s}\in \left\{4\mathrm{f}\right\},\ \mathrm{d}\left(\mathrm{s}\right)\in \left\{1\right\},\hfill \end{array} $$

where in terms of the independent exponential random variables {*H*_4*f*,*d*(4*f*),*j*_ : *j* = {1, 2, 3, 6}} used to define *H*_4*f*,*d*(4*f*)_, we define the auxiliary random variable

2f $$ Z= \min\ \left\{{H}_{4f,d(4f),j}:\ j=\left\{1,\ 2,\ 3,\ 6\right\}\right\} $$

which is exponential with rate parameter

2g $$ {\lambda}_Z={\displaystyle {\sum}_{j\in \left\{1,\ 2,\ 3,\ 6\right\}}{\lambda}_{4f,d(4f),j}}. $$

In Eq. (2b–e), the state-dependent nurse time, *γ*_*nurse*_(*s*) is computed as the additional time in hours exceeding the baseline nurse time for a patient in state *5*. The term *γ*_*RRT*_(*s*) represents the time that the RRT spends in moving to/from the patient’s room and onsite treating the patient if the RRT is activated at a decision epoch, measured in hours. The indicator variable, *c*(*s*, *j*) accumulates TTS or FTR. The variable, *c*(*s*, *j*) takes the value one if the state transition from s to *j* corresponds to TTS or FTR; otherwise it takes the value of zero. Table 18 summarizes the values of *c*(*s*, *j*).

**Table 18:** Values of indicator variable *c*(*s*, *j*) where *s* is the current state and *j* is the subsequent state

s / j	5	4s	4f	3	2	1	6
5	0	0	0	0	0	0	0
4s	0	0	0	0	0	0	0
4f	0	1	0	1	1	1	1
3	0	0	1	0	1	1	1
2	0	0	1	1	0	1	1
1	0	0	1	1	1	0	1
6	0	0	0	0	0	0	0

The quantity *u* is used as the variable of integration. Equation (2a) represents the case if the state at the current decision epoch is *s* ∈ {5, 4 *s*}. Equation (2b) represents a patient in state *s* ∈ {3, 2} at the current decision epoch with TTS (recovery) accumulation. Equation (2c) represents a patient in distress state 1 at the current decision epoch with TTS (recovery), and possible FTR accumulation. Equation (2d) represents a patient in distress state 1 at the current decision epoch with TTS (recovery) accumulation without FTR (because RRT is activated). Equation (2e) is the case where the patient is in state 4*f* at the current decision epoch with possible TTS accumulation (unsuccessful stabilization if Z<1 and successful stabilization otherwise).

In the modified SMDP model, the weight *p*(*s*) is incorporated in the function, *r*′(*s*, *d*(*s*)), given the current state *s* and action *d* (*s*):

3a $$ {r}^{\prime}\left(s,d(s)\right)=\rho (s)r\left(s,d(s)\right) + \left(1-\rho (s)\right)\overline{r}\left(s,d(s)\right) $$

where *r*(*s*, *d*(*s*)) is defined in Eq. (2a–e). The term $\overline{r}\left(s,d(s)\right)$ is the function, where ${\overline{\gamma}}_{nurse}(s)= \max \left\{{\gamma}_{nurse}(s):s\in S\right\}$ and ${\overline{\gamma}}_{RRT}(s)= \max \left\{{\gamma}_{RRT}(s):s\in S\right\}$ as observed in the electronic medical records. The function $\overline{r}\left(s,d(s)\right)$ is given by:

3b $$ \overline{r}\left(s,d(s)\right)={\overline{\gamma}}_{\mathrm{nurse}}(s)\kern0.75em \mathrm{f}\mathrm{o}\mathrm{r}\ \mathrm{s}\in \left\{5,\ 4\mathrm{s}\right\},\mathrm{d}\left(\mathrm{s}\right)=\left\{1\right\}, $$

3c $$ \begin{array}{l}\overline{r}\left(s,d(s)\right)={\overline{\gamma}}_{\mathrm{nurse}}(s)+I\left(d(s)=2\right){\overline{\gamma}}_{\mathrm{RRT}}(s)\hfill \\ {}+{\displaystyle {\int}_0^{\infty}\left[{\displaystyle {\int}_0^u{e}^{-\alpha t}c\left(s,4f\right)P\left(4f|s,d(s)\right)dt}\right]}{\lambda}_{s,d(s)}{e}^{-{\lambda}_{s,d(s)}u}du\hfill \\ {}\mathrm{f}\mathrm{o}\mathrm{r}\ \mathrm{s}\in \left\{2,\ 3\right\},\ \mathrm{j}=\left\{4\mathrm{f}\right\},\ \mathrm{d}\left(\mathrm{s}\right)\in \left\{1,\ 2\right\},\hfill \end{array} $$

3d $$ \begin{array}{l}\overline{r}\left(s,d(s)\right)={\overline{\gamma}}_{\mathrm{nurse}}(s)\hfill \\ {}+{\displaystyle {\int}_0^{\infty}\left[{\displaystyle {\int}_0^u{e}^{-\alpha t}c\left(s,4f\right)P\left(4f|s,d(s)\right)dt}\right]}{\lambda}_{s,d(s)}{e}^{-{\lambda}_{s,d(s)}u}du\hfill \\ {}+{\displaystyle {\int}_1^{\infty }{\displaystyle {\sum}_{j\in S}\left[{\displaystyle {\int}_0^u{e}^{-\alpha t}c\left(s,j\right)P\left(j|s,d(s)\right)dt}\right]}}{\lambda}_{s,d(s)}{e}^{-{\lambda}_{s,d(s)}u}du\hfill \\ {}\mathrm{f}\mathrm{o}\mathrm{r}\ \mathrm{s}\in \left\{1\right\},\ \mathrm{j}\in \mathrm{S},\ \mathrm{d}\left(\mathrm{s}\right)=\left\{1\right\},\hfill \end{array} $$

3e $$ \begin{array}{l}\overline{r}\left(s,d(s)\right)={\overline{r}}_{\mathrm{nurse}}(s)+{\overline{\gamma}}_{\mathrm{RRT}}(s)\hfill \\ {}+{\displaystyle {\int}_0^{\infty}\left[{\displaystyle {\int}_0^u{e}^{-\alpha t}c\left(s,4f\right)P\left(4f|s,d(s)\right)dt}\right]}{\lambda}_{s,d(s)}{e}^{-{\lambda}_{s,d(s)}u}du\hfill \\ {}\mathrm{f}\mathrm{o}\mathrm{r}\ \mathrm{s}\in \left\{1\right\},\ \mathrm{j}\in \mathrm{S},\ \mathrm{d}\left(\mathrm{s}\right)=\left\{2\right\},\hfill \end{array} $$

3f $$ \begin{array}{l}\overline{r}\left(s,d(s)\right)={\overline{\gamma}}_{\mathrm{nurse}}(s)\hfill \\ {}+0{\displaystyle {\int}_0^1{\displaystyle {\sum}_{j\in \left\{1,2,3,6\right\}}P\left(j|4f,\ d(4f),Z<1\right)}}\left[{\displaystyle {\int}_0^u{e}^{-\alpha t}c\left(4f,j\right)dt}\right]{\lambda}_Z{e}^{-{\lambda}_Zu}du\hfill \\ {} + {e}^{-{\lambda}_Z}{\displaystyle {\int}_0^1{e}^{-\alpha t}c\left(4f,4s\right)dt}\hfill \\ {}\mathrm{f}\mathrm{o}\mathrm{r}\ \mathrm{s}\in \left\{4\mathrm{f}\right\},\ \mathrm{d}\left(\mathrm{s}\right)\in \left\{1\right\}.\hfill \end{array} $$

## APPENDIX 3: Time to stabilization (TTS)-related times in state 4*f*

We define a random variable *Z*≡ min{*H*_4*f*,*d*(4*f*),*j*_ : *j* = 1, 2, 3, 6}. Therefore, *Z* follows an exponential distribution with rate *λ*_*Z*_ = ∑_*j* ∈ {1,2,3,6}_ *λ*_4*f*,*d*(4*f*),*j*_. The sojourn time in state 4*f* is defined as

$$ {H}_{4f,d(4f)}=\left\{\begin{array}{c}\hfill Z,\kern0.75em \mathrm{if}\ Z<1,\hfill \\ {}\hfill 1,\kern0.75em \mathrm{if}\ Z\ge 1.\hfill \end{array}\right. $$

Given *Z* < 1, the conditional PDF of *H*_4*f*,*d*(4*f*)_ is given by

$$ {f}_{4f,d(4f)}(u)=\left\{\begin{array}{c}\hfill 0,\kern1.25em \mathrm{if}\ u<0,\hfill \\ {}\hfill \frac{\lambda_Z{e}^{-{\lambda}_Zu}}{\left(1-{e}^{-{\lambda}_Z}\right)},\ \mathrm{if}\ 0\le u<1,\hfill \\ {}\hfill 1,\kern1.5em \mathrm{if}\ u\ge 1.\hfill \end{array}\ \right. $$

Let *J* be the state to which the process jumps when it leaves the state 4*f*. It follows that:

$$ \begin{array}{l}P\ \left\{j|4f,\ d(4f),\ Z<1\right\}=P\left\{J=j\ |Z<1\right\}\ \\ {}=P\left\{{H}_{4f,d(4f),j}<{H}_{4f,d(4f),k}\kern0.5em \mathrm{f}\mathrm{o}\mathrm{r}\ k=\left\{1,\ 2,\ 3,\ 6\right\}\backslash \left\{j\right\}\ |\ Z<1\right\}\kern0.5em \mathrm{f}\mathrm{o}\mathrm{r}\ j\in \left\{1,\ 2,\ 3,\ 6\right\}\end{array} $$

To simplify the notation, let *L*={1,2,3,6} and for *j* ∈ *L*, let *L*_*j*_=*L*\{*j*}. Then, for *j* ∈ *L*

4a $$ \begin{array}{l}P\left\{j\ |\ 4f,d(4f),Z<1\right\}\\ {}=P\left\{Z={H}_{4f,d(4f),j},\ Z<{H}_{4f,d(4f),k}\ \mathrm{f}\mathrm{o}\mathrm{r}\ k\in {L}_j\ |\ Z<1\right\}\end{array} $$

4b $$ =\frac{P\left\{Z={H}_{4f,d(4f),j},\kern0.75em Z<{H}_{4f,d(4f),k}\ \mathrm{f}\mathrm{o}\mathrm{r}\ k\in {L}_j,\kern0.75em Z<1\right\}}{P\left\{Z<1\right\}} $$

Conditioning on *H*_4*f*,*d*(4*f*),*j*_ = *u*, we have:

4c $$ \begin{array}{l}={\left(1-{e}^{-{\lambda}_Z}\right)}^{-1}{\displaystyle {\int}_0^1P\left\{Z={H}_{4f,d(4f),j},\ Z<{H}_{4f,d(4f),k}\ \mathrm{f}\mathrm{o}\mathrm{r}\ k\in {L}_j,\ Z<1\ \Big|\ {H}_{4f,d(4f),j}=u\right\}}\cdot \\ {}{\lambda}_{4f,d(4f),j}\cdot {e}^{-{\lambda}_{4f,d(4f),j \cdot u}}\ du\end{array} $$

4d $$ ={\left(1-{e}^{-{\lambda}_Z}\right)}^{-1}{\displaystyle {\int}_0^1P\left\{u<{H}_{4f,d(4f),k}\ \mathrm{f}\mathrm{o}\mathrm{r}\ k\in {L}_j\right\}\cdot {\lambda}_{4f,d(4f),j}\cdot {e}^{-{\lambda}_{4f,d(4f),j \cdot u}}\ du} $$

4e $$ ={\left(1-{e}^{-{\lambda}_Z}\right)}^{-1}{\displaystyle {\int}_0^1{e}^{-u{\displaystyle {\sum}_{k\in {L}_j}}{\lambda}_{4f,d(4f),k}} \cdot {\lambda}_{4f,d(4f),j}\cdot {e}^{-{\lambda}_{4f,d(4f),j \cdot u}}\ du} $$

4f $$ ={\left(1-{e}^{-{\lambda}_Z}\right)}^{-1}{\displaystyle {\int}_0^1{e}^{-{\lambda}_Zu} \cdot {\lambda}_{4f,d(4f),j}\ du} $$

because we have

4g $$ {\lambda}_Z={\displaystyle {\sum}_{k\in L}{\lambda}_{4f,d(4f),k}}={\lambda}_{4f,d(4f),j} + {\displaystyle {\sum}_{k\in {L}_j}{\lambda}_{4f,d(4f),k}}. $$

Thus, from expression (4f) we have

$$ P\left\{j\ \Big|\ 4f,d(4f),Z<1\right\}=\frac{\lambda_{4f,d(4f),j\ }}{\lambda_Z}\cdot {\left(1-{e}^{-{\lambda}_Z}\right)}^{-1}{\displaystyle {\int}_0^1{\lambda}_Z{e}^{\lambda_Zu}du=\frac{\lambda_{4f,d(4f),j\ }}{\lambda_Z}}. $$ (4h)

For the expression (4h), we have

$$ {\displaystyle {\sum}_{j\in L}P\left\{j|4f,d(4f),Z<1\right\}=1} $$

Let *G*_*TTS*_(*Z*, *J*) denote the following:

- when *Z* < 1, *G*_*TTS*_(*Z*, *J*) denotes the rate at which TTS-related time is accumulated over the random time interval [O,Z] and is expressed as a function of the random variables *Z* and *J*,
- when *Z* = 1, *G*_*TTS*_(*Z*, *J*) denotes the rate at which TTS-related time is accumulated over the fixed time interval [0, 1]; and in this case the destination state is fixed *J* = 4*s*, denoting the stabilization of a patient.

We formulate *G*_*TTS*_(*Z*, *J*) as

$$ {G}_{TTS}\left(Z,J\right)=\left\{\begin{array}{c}\hfill c\left(4f,J\right)\kern1.25em \mathrm{if}\ Z<1,\hfill \\ {}\hfill c\left(4f,4s\right)\kern0.75em \mathrm{if}\ Z=1.\hfill \end{array}\right. $$

The total discounted TTS-related time is given by

$$ {G}_{TTS}^{*}\left(Z,J\right)=\left\{\begin{array}{c}\hfill {\displaystyle {\int}_0^Z{e}^{-\alpha t}c\left(4f,J\right)dt\kern1em \mathrm{if}\ Z<1,}\hfill \\ {}\hfill {\displaystyle {\int}_0^1{e}^{-\alpha t}c\left(4f,4s\right)dt\kern1em \mathrm{if}\ Z=1.}\hfill \end{array}\right. $$

The expectation is computed as follows:

$$ \begin{array}{l}E\left[{G}_{TTS}^{*}\left(Z,J\right)\right]=P\left\{Z<1\right\}E\left[{G}_{TTS}^{*}\left(Z,J\right)\ |\ Z<1\right]+P\left\{Z=1\right\}E\left[{G}_{TTS}^{*}\left(Z,J\right)\ |\ Z=1\right]\\ {}=\left(1-{e}^{-{\lambda}_Z}\right)E\left[{\displaystyle {\int}_0^Z{e}^{-\alpha t}c\left(4f,J\right)dt\ |\ Z<1}\right]+{e}^{-{\lambda}_Z}E\left[{\displaystyle {\int}_0^Z{e}^{-\alpha t}c\left(4f,4s\right)dt\ |Z=1}\right]\\ {}=\left(1-{e}^{-{\lambda}_Z}\right){\displaystyle {\int}_0^1{\displaystyle {\sum}_{j\in \left\{1,\ 2,\ 3,\ 6\right\}}P\left(j|4f,\ d(4f),Z<1\right)}}\left[{\displaystyle {\int}_0^u{e}^{-\alpha t}c\left(4f,j\right)dt}\right]\left[\frac{\lambda_Z{e}^{-{\lambda}_Zu}}{1-{e}^{-{\lambda}_Z}}\right]du\\ {} + {e}^{-{\lambda}_Z}{\displaystyle {\int}_0^1{e}^{-\alpha t}c\left(4f,4s\right)dt.}\end{array} $$

The first integral in (4i) is given by

5a $$ \left(1-{e}^{-{\lambda}_Z}\right){\displaystyle {\int}_0^1\left\{{\displaystyle {\sum}_{j\in L}\frac{\lambda_{4f,d(4f),j}}{\lambda_Z}\cdot \frac{c\left(4f,j\right)}{\alpha}\cdot \left(1-{e}^{-\alpha u}\right)\cdot \frac{\lambda_Z{e}^{-{\lambda}_Zu}}{1-{e}^{-{\lambda}_Z}}}\right\}}du $$

5b $$ ={\displaystyle {\sum}_{j\in L}\frac{\lambda_{4f,d(4f),j} \cdot c\left(4f,j\right)}{\alpha}\cdot \left\{{\displaystyle {\int}_0^1\left(1-{e}^{-\alpha u}\right)\cdot {e}^{-{\lambda}_Zu}\ du}\ \right\}} $$

5c $$ ={\displaystyle {\sum}_{j\in L}\frac{\lambda_{4f,d(4f),j} \cdot c\left(4f,j\right)}{\alpha}\cdot \left\{{\displaystyle {\int}_0^1\ {e}^{-{\lambda}_Zu}du-{\displaystyle {\int}_0^1{e}^{-\left({\lambda}_Z+\alpha \right)}du}}\right\}} $$

5d $$ ={\displaystyle {\sum}_{j\in}\frac{\lambda_{4f,d(4f),j} \cdot c\left(4f,j\right)}{\alpha}\cdot \left\{\frac{1-{e}^{-{\lambda}_Z}}{\lambda_Z}-\frac{1-{e}^{-\left({\lambda}_Z+\alpha \right)}}{\lambda_Z+\alpha}\right\}} $$

5e $$ ={\displaystyle {\sum}_{j\in L}\frac{\lambda_{4f,d(4f),j} \cdot c\left(4f,j\right)\cdot \left[\alpha -\left({\lambda}_Z+\alpha \right){e}^{-{\lambda}_Z}+{\lambda}_Z{e}^{-\left({\lambda}_Z+\alpha \right)}\right]}{\alpha \cdot {\lambda}_Z\cdot \left({\lambda}_Z+\alpha \right)}} $$

The second integral in (4i) is given by

5f $$ {e}^{-{\lambda}_Z}\cdot c\left(4f,4s\right)\cdot {\displaystyle {\int}_0^1{e}^{-\alpha t}dt} $$

5g $$ =\frac{e^{-{\lambda}_Z}\cdot c\left(4f,4s\right)\cdot \left[1-{e}^{-\alpha}\right]}{\alpha } $$

5h $$ =\frac{c\left(4f,4s\right)\cdot \left[{e}^{-{\lambda}_Z}-{e}^{-\left({\lambda}_Z+\alpha \right)}\right]}{\alpha } $$

Combining (5e) and (5h), we have

5i $$ E\left[{G}_{TTS}^{*}\left(Z,J\right)\right]=\left[{\displaystyle {\sum}_{j\in L}\frac{\lambda_{4f,d(4f),j} \cdot c\left(4f,j\right)\cdot \left[\alpha -\left({\lambda}_Z+\alpha \right){e}^{-{\lambda}_Z}+{\lambda}_Z{e}^{-\left({\lambda}_Z+\alpha \right)}\right]}{\alpha \cdot {\lambda}_Z\cdot \left({\lambda}_Z+\alpha \right)}}\right] + \frac{c\left(4f,4s\right)\cdot \left[{e}^{-{\lambda}_Z}-{e}^{-\left({\lambda}_Z+\alpha \right)}\right]}{\alpha } $$

## APPENDIX 4: Failure to rescue (FTR)-related time in model state 1

Failure to rescue (FTR) is only applicable in clinical distress state (state 1) with action *d*(1) = 1 (no RRT activation). Let *U* denote the sojourn time in state *1*, and let *J* denote the state to which the process moves when it leaves state 1. Let *G*_*FTR*_(*U*, *J*) denote the rate at which the FTR-related time is accumulated given by

$$ {G}_{FTR}\left(U,J\right)=\left\{\begin{array}{c}\hfill c\left(1,J\right)\kern0.5em  if\ U\ge 1,\hfill \\ {}\hfill 0\kern2.75em  if\ U<1.\hfill \end{array}\right. $$

The discounted total FTR-related time is given by

$$ {G^{*}}_{FTR}\left(U,J\right)={\displaystyle {\int}_0^U{e}^{-\alpha t}{G}_{FTR}\left(U,J\right)dt}. $$

Then, the expected value of the total discounted FTR-related time is

$$ \begin{array}{c}\hfill E\left[{G^{*}}_{FTR}\left(U,J\right)\right]={\displaystyle {\int}_0^{\infty }{\displaystyle \sum_{j\in S}}E\left[{G^{*}}_{FTR}\left(U,J\right)\ \Big|\ U=u,\ J=j\right]P\left(j\Big|1,d(1)=1\right){\lambda}_{1,d(1)=1}{e}^{-{\lambda}_{1,d(1)=1}u}du}\hfill \\ {}\hfill ={\displaystyle {\int}_0^{\infty }{\displaystyle {\sum}_{j\in S}P\left(j\Big|1,\ d(1)=1\right)\left[{\displaystyle {\int}_0^u{e}^{-\alpha t}{G}_{FTR}\left(u,j\right)dt}\right]{\lambda}_{1,d(1)=1}{e}^{-{\lambda}_{1,d(1)=1}u}du}}\hfill \\ {}\hfill ={\displaystyle {\int}_1^{\infty }{\displaystyle {\sum}_{j\in S}P\left(j\Big|1,d(1)=1\right)\left[{\displaystyle {\int}_0^u{e}^{-\alpha t}c\left(1,j\right)dt}\right]{\lambda}_{1,d(1)=1}{e}^{-{\lambda}_{1,d(1)=1}u}du}}\hfill \\ {}\hfill ={\displaystyle {\sum}_{j\in S}P\left(j\Big|1,\ d(1)=1\right)\frac{c\left(1,j\right)}{\alpha }{\displaystyle {\int}_1^{\infty}\left(1-{e}^{-\alpha u}\right){\lambda}_{1,d(1)=1}{e}^{-{\lambda}_{1,d(1)=1}u}du}}\hfill \\ {}\hfill ={\displaystyle {\sum}_{j\in S}P\left(j\Big|1,\ d(1)=1\right)\frac{c\left(1,j\right)}{\alpha }{\displaystyle {\int}_1^{\infty}\left(1-{e}^{-\alpha u}\right){\lambda}_{1,d(1)=1}{e}^{-{\lambda}_{1,d(1)=1}u}du}}\ \hfill \end{array} $$

## APPENDIX 5: Baseline and modified SMDP model results for medical patients with moderate ROD

The table below summarizes the optimal value function in state *s*, *v*(*s*), representing the total expected stabilization related time in hours and optimal state-dependent RRT activation policy, *d**(*s*), for *s* ∈ *S* in the baseline SMDP model, and modified SMDP model under three Baseline and five Modified Scenarios.

**Table 19:** Baseline SMDP results

Optimal value function [hrs.]						Optimal policy d*(s)					
v(5)	v(4s)	v(4f)	v(3)	v(2)	v(1)	d*(5)	d*(4s)	d*(4f)	d*(3)	d*(2)	d*(1)
6.66	7.30	9.46	10.69	12.38	12.79	Wait			Call		

**Table 20:** Baseline scenario #1 results, clustering algorithm: single linkage

	Optimal value function [hrs.]						Optimal policy d*(s)					
Cluster #	v(5)	v(4s)	v(4f)	v(3)	v(2)	v(1)	d*(5)	d*(4s)	d*(4f)	d*(3)	d*(2)	d*(1)
1	10.22	11.37	11.47	12.50	15.66	15.67	Wait			Call		
2	10.59	11.05	11.16	11.88	14.57	16.24						Wait
3	10.55	10.97	11.08	12.22	15.14	15.79						
4	11.20	12.42	12.50	13.90	15.26	15.53						

**Table 21:** Baseline scenario #2 results, clustering algorithm: Ward’s method

	Optimal value function [hrs.]						Optimal policy d*(s)					
Cluster #	v(5)	v(4s)	v(4f)	v(3)	v(2)	v(1)	d*(5)	d*(4s)	d*(4f)	d*(3)	d*(2)	d*(1)
1	10.22	11.37	11.47	12.50	15.66	15.67	Wait					
2	10.59	11.05	11.16	11.88	14.57	16.24						Wait
3	11.02	11.52	11.61	12.39	16.51	16.20				Call		
4	11.25	11.28	11.38	12.85	14.98	15.68						
5	10.44	11.98	12.06	13.07	16.05	17.08						
6	10.55	11.13	11.24	12.20	15.71	15.80						
7	9.13	10.55	10.67	11.97	14.28	14.36						

**Table 22:** Baseline scenario #3 results, clustering algorithm: centroid

	Optimal value function [hrs.]						Optimal policy d*(s)					
Cluster #	v(5)	v(4s)	v(4f)	v(3)	v(2)	v(1)	d*(5)	d*(4s)	d*(4f)	d*(3)	d*(2)	d*(1)
1	11.22	11.37	11.47	12.50	15.66	15.67	Wait					
2	10.59	11.05	11.16	11.88	14.57	16.24						Wait
3	11.23	11.67	11.76	12.45	16.43	16.79				Call		
4	11.44	11.98	12.06	13.07	16.05	17.08						
5	10.91	11.55	11.65	12.63	16.57	16.04						
6	10.29	10.71	10.83	12.08	14.60	14.53						

**Table 23:** Modified scenario #1 results, clustering algorithm: single linkage

	Optimal value function [hrs.]						Optimal policy d*(s)					
Cluster #	v(5)	v(4s)	v(4f)	v(3)	v(2)	v(1)	d*(5)	d*(4s)	d*(4f)	d*(3)	d*(2)	d*(1)
1	11.18	11.30	11.40	11.47	16.31	16.52	Wait					
2	9.06	9.46	9.61	10.01	11.97	14.73						Wait
3	11.10	11.35	11.45	12.67	15.42	15.55				Call		
4	12.05	12.57	12.64	13.79	16.94	17.69						
5	10.45	10.73	10.85	11.49	15.00	15.73						

**Table 24:** Modified scenario #2 results, clustering algorithm: single linkage

	Optimal value function [hrs.]						Optimal policy d*(s)					
Cluster #	v(5)	v(4s)	v(4f)	v(3)	v(2)	v(1)	d*(5)	d*(4s)	d*(4f)	d*(3)	d*(2)	d*(1)
1	11.64	11.98	12.06	13.16	15.75	17.37	Wait					
2	11.19	11.65	11.75	13.18	14.15	16.41						Wait
3	11.75	12.36	12.43	13.84	17.58	17.91				Call		
4	11.29	11.70	11.79	14.28	15.27	15.58						
5	10.61	11.08	11.19	12.58	15.20	15.82						

**Table 25:** Modified scenario #3 results, clustering algorithm: single linkage

	Optimal value function [hrs.]						Optimal policy d*(s)					
Cluster #	v(5)	v(4s)	v(4f)	v(3)	v(2)	v(1)	d*(5)	d*(4s)	d*(4f)	d*(3)	d*(2)	d*(1)
1	10.34	10.90	11.01	11.41	14.69	15.95	Wait					
2	11.47	11.98	12.07	12.09	14.87	17.07						Wait
3	11.32	12.15	12.23	13.23	16.70	16.82				Call		
4	12.11	12.53	12.60	13.17	17.26	17.78						
5	11.03	11.87	11.96	14.70	14.72	16.58						
6	10.30	10.63	10.75	11.34	14.91	14.96						

**Table 26:** Modified scenario #4 results, clustering algorithm: single linkage

	Optimal value function [hrs.]						Optimal policy d*(s)					
Cluster #	v(5)	v(4s)	v(4f)	v(3)	v(2)	v(1)	d*(5)	d*(4s)	d*(4f)	d*(3)	d*(2)	d*(1)
1	7.82	8.49	8.67	9.29	11.50	11.92	Wait					
2	6.24	6.97	7.18	7.75	8.92	10.31						Wait
3	8.36	8.05	8.23	8.52	11.02	11.44				Call		
4	8.47	9.12	9.28	10.37	12.18	12.60						

**Table 27:** Modified scenario #5 results, clustering algorithm: single linkage

	Optimal value function						Optimal policy					
Cluster #	v(5)	v(4s)	v(4f)	v(3)	v(2)	v(1)	d*(5)	d*(4s)	d*(4f)	d*(3)	d*(2)	d*(1)
1	9.76	10.13	10.26	11.35	13.65	14.01	Wait			Call		
2	10.41	11.07	11.18	12.86	15.11	15.56						
3	9.63	10.13	10.26	11.37	13.78	13.82						
